# Effects of Long-Term Salinity Exposure on Growth, Survival, Hepatopancreatic Health, and Intestinal Microbiota in Juvenile Chinese Mitten Crabs (*Eriocheir japonica sinensis*)

**DOI:** 10.3390/ani16203192

**Published:** 2026-10-11

**Authors:** Xiang Lu, Dan-Dan Bian, Xin Liu, Xin-Yao An, Bo-Ping Tang, Qiu-Ning Liu

**Affiliations:** 1 College of Fisheries and Life Science, Shanghai Ocean University, Shanghai 201306, China; luxiangshou@163.com (X.L.); m220150485@st.shou.edu.cn (X.L.); anxinyao0719@163.com (X.-Y.A.); 2 College of Life Sciences, Anhui Agricultural University, Hefei 230036, China; biandd1354@163.com; 3 Jiangsu Key Laboratory for Bioresources of Saline Soils, Jiangsu Synthetic Innovation Center for Coastal Bio-Agriculture, Jiangsu Provincial Key Laboratory of Coastal Wetland Bioresources and Environmental Protection, School of Wetlands, Yancheng Teachers University, Yancheng 224007, China

**Keywords:** *Eriocheir japonica sinensis*, salinity tolerance, hepatopancreas, multi-omics, intestinal microbiota

## Abstract

Salinity is a key environmental factor in crustacean aquaculture, but its long-term effects on juvenile Chinese mitten crabs (*Eriocheir japonica sinensis*) remain poorly understood. Megalopae were reared for seven months at 0, 15, and 30 ppt, with three replicate tanks per treatment. Growth, survival, hepatopancreatic histology and biochemistry, multi-omics profiles, and intestinal bacterial communities were evaluated. Crabs at 0 ppt achieved the greatest growth and production. Survival was highest at 15 ppt, although growth and production were lowest, whereas survival was lowest at 30 ppt despite greater growth among survivors than at 15 ppt. Increasing salinity was associated with more pronounced hepatopancreatic lesions and TUNEL-positive signals, increased serum aminotransferase activities, and decreased albumin. Immune-related enzyme activities increased under saline conditions, whereas antioxidant capacity declined and lipid peroxidation increased. Multi-omics analyses highlighted the Lysosome and Tyrosine metabolism pathways. Intestinal bacterial diversity tended to increase with salinity, accompanied by a greater relative abundance of *Vibrio* and a lower abundance of *Dysgonomonas*. Thus, 15 ppt favored survival without supporting growth, whereas 30 ppt was associated with the poorest tissue condition and lowest survival. These findings show that survival, growth, production, and physiological health should be considered together when evaluating salinity suitability for long-term culture.

## Simple Summary

Chinese mitten crabs encounter waters of different salinities during their life cycle and may also be farmed in brackish or saline areas. However, little is known about how prolonged salinity exposure affects juvenile growth and health. We reared juvenile crabs in freshwater or at salinities of 15 and 30 parts per thousand for seven months and compared their growth, survival, hepatopancreatic condition, and intestinal bacteria. The hepatopancreas supports digestion, nutrient storage, and immune defense. Crabs reared in freshwater showed the greatest growth and harvest yield. Crabs reared at 15 parts per thousand had the highest survival but the lowest growth and yield. Crabs reared at 30 parts per thousand had the lowest survival, more extensive hepatopancreatic damage, weaker antioxidant protection, and a greater relative abundance of *Vibrio* bacteria, although the surviving crabs grew better than those at 15 parts per thousand. Analyses of genes and small molecules also showed changes in pathways related to cellular maintenance and amino acid use. These findings show that high survival does not necessarily indicate optimal growth or health. Salinity management should therefore consider survival, production, and organ condition together when evaluating water sources for the long-term culture of juvenile Chinese mitten crabs.

## 1. Introduction

Salinity is a major environmental factor influencing the survival, growth, and physiological condition of aquatic animals, particularly euryhaline crustaceans that encounter different salinity regimes during their life cycles [1,2]. In decapod crustaceans, salinity fluctuations challenge osmotic and ionic balance and require energy-demanding adjustments in osmoregulation, metabolism, antioxidant defenses, and innate immune activity [3,4]. When these demands persist, redox balance may be disturbed, leading to the accumulation of reactive oxygen species, cellular damage, and impaired tissue function [5,6]. These effects are relevant to aquaculture because chronic physiological stress can adversely affect animal health and production performance [7]. Salinity can also shape intestinal bacterial communities through its effects on both environmental microorganisms and the host intestinal environment [8,9]. However, responses observed during acute exposure may not adequately predict physiological acclimation, cumulative injury, or production trade-offs over longer rearing periods. For juvenile Chinese mitten crab, it remains unclear whether salinity maintained over an extended rearing cycle produces parallel or divergent responses in growth, survival, hepatopancreatic condition, and intestinal bacterial communities. This knowledge gap limits the reliability of evaluating salinity suitability for long-term culture based on survival or growth alone.

The Chinese mitten crab (*Eriocheir japonica sinensis*) is an economically important catadromous crustacean. Adults migrate from freshwater habitats to brackish or marine waters for reproduction, whereas juveniles subsequently migrate into freshwater environments for growth [10,11,12]. The Yangtze River estuary encompasses a broad freshwater-to-marine gradient, with salinity ranging from approximately 0 to 30 ppt among different areas. During recruitment, megalopae and newly metamorphosed juveniles move from saline estuarine waters toward freshwater habitats. Previous monitoring during a seawater-to-freshwater transition documented a decline in salinity from 20 to 10 practical salinity units (PSU), including an intermediate salinity of 15 PSU [13,14]. Thus, although freshwater is the principal grow-out habitat of juvenile crabs, their early life stages may encounter substantial changes in salinity during migration. This life cycle indicates a broad capacity for salinity acclimation but does not preclude physiological costs associated with prolonged exposure to non-optimal salinities. Previous studies have demonstrated that salinity affects osmoregulatory gene expression, tissue morphology, antioxidant capacity, and metabolism in *E. j. sinensis* [15,16,17,18]. Although long-term salinity adaptation has been examined in adults [16], evidence for juvenile crabs over an extended rearing period remains limited. Long-term exposure may involve both physiological acclimation and the cumulative effects of sustained osmotic and energetic demands. Evaluating growth and survival alongside health-related responses is therefore necessary to distinguish physiological acclimation from cumulative stress in juvenile *E. j. sinensis* during prolonged rearing.

The hepatopancreas is central to digestion, nutrient storage, metabolism, detoxification, and immune-related functions in crustaceans, and its structural and functional integrity is an important indicator of animal health [19,20,21]. Nonspecific immune status is commonly evaluated from the activities of acid phosphatase (ACP), alkaline phosphatase (AKP), and lysozyme (LZM) [2,5,6]. Antioxidant status can similarly be characterized using superoxide dismutase (SOD), catalase (CAT), glutathione peroxidase (GSH-Px), and total antioxidant capacity (T-AOC), together with malondialdehyde (MDA) as an indicator of lipid peroxidation [6]. Beyond these biochemical responses, autophagy and apoptosis play distinct roles in cellular responses to environmental stress [22,23,24,25]. Autophagy supports cellular homeostasis by delivering damaged proteins and organelles to lysosomes for degradation, whereas apoptosis may be activated when cellular damage is severe or persistent [26,27]. Autophagy is regulated by energy- and stress-responsive signaling networks. AMPK links cellular energy status to the regulation of autophagy [22,23], whereas FoxO can couple redox signals to the transcriptional control of autophagy-related genes [24]. Molecular changes in these pathways may therefore provide insight into cellular maintenance and injury under stress. However, it remains unclear how prolonged salinity exposure affects these responses and how they relate to hepatopancreatic damage in juvenile *E. j. sinensis*.

The intestinal bacterial community provides a complementary perspective on the effects of salinity. Intestinal microorganisms contribute to nutrient utilization, epithelial function, and immune regulation in aquatic animals, while their composition is sensitive to environmental conditions [28,29,30]. Environmental factors, including salinity, can alter intestinal bacterial diversity and community composition and may favor shifts in bacterial taxa associated with stressed aquaculture animals [7,8,31]. Salinity-induced shifts in bacterial taxa of aquaculture relevance, including members of *Vibrio*, may be particularly important when they occur alongside impaired host condition [31,32,33]. Previous studies have suggested associations between intestinal bacterial communities and hepatopancreatic or hepatic function in aquatic animals [34,35,36]. Therefore, examining hepatopancreatic responses and intestinal microbiota together may help determine whether microbial changes accompany the physiological effects of long-term salinity exposure.

Accordingly, this study examined the long-term effects of salinity on the performance and health of juvenile *E. j. sinensis*. The specific objectives were to (1) compare growth, survival, and production among salinity treatments; (2) determine whether these performance differences were accompanied by changes in hepatopancreatic structure, biochemical condition, and molecular pathways; and (3) characterize changes in the intestinal bacterial community and determine whether they coincided with differences in animal performance and hepatopancreatic condition. We hypothesized that growth and survival would respond differently across salinity levels and that these responses would be accompanied by changes in hepatopancreatic condition and intestinal bacterial community composition. By assessing growth and survival together with hepatopancreatic injury and intestinal bacterial community structure, this study goes beyond evaluating salinity suitability based on production outcomes alone. This combined assessment helps distinguish the ability of juvenile crabs to survive at a given salinity from the overall suitability of that salinity for long-term culture, which also depends on sustained growth and production and the maintenance of tissue health.

## 2. Materials and Methods

### 2.1. Animal Ethics Statement

This study was approved by the Institutional Animal Care and Use Committee (IACUC) of the Shanghai Ocean University (SHOU), Shanghai, China (SHOU-DW-2016-004).

### 2.2. Experimental Animals, Rearing Design, and Sampling Strategy

Experimental megalopae were obtained from Jinyang Aquatic Germplasm Resources Field (Yancheng, Jiangsu Province, China), where the rearing experiment was conducted in an indoor aquaculture facility (E 120°38′, N 33°50′) from 15 May to 15 December. Before transfer to the experimental tanks, the megalopae underwent a two-day salinity-reduction procedure. They were initially held in a desalination pond containing seawater, after which freshwater was progressively added while water was discharged, reducing the salinity to approximately 7 ppt by the end of the two-day period. No additional acclimation to the experimental salinities was conducted after this procedure. All tanks had been adjusted to their target salinities before stocking, and experimental exposure began when the megalopae were introduced into their assigned tanks. According to information provided by the supplier, their mean initial wet weight was approximately 0.0065 g. Nine independent cement tanks (6 m × 4 m × 1.5 m) were randomly assigned to three salinity treatments (0, 15, and 30 ppt), with three tanks per treatment. Each tank served as an independent experimental unit and was stocked with 40 g of megalopae, corresponding to approximately 6154 individuals. Sex was not determined at stocking because external sexual characteristics cannot be reliably distinguished at the megalopa stage, and sex composition was not recorded at harvest; sex was therefore not included as a factor in the analysis. After the seven-month rearing period, the animals were regarded as juvenile crabs, but gonadal maturity was not assessed.

Groundwater was used for the 0 ppt treatment. Natural seawater obtained from coastal waters adjacent to the facility was settled and filtered in an outdoor reservoir before use. The 15 ppt treatment was prepared by mixing groundwater with treated seawater, whereas treated seawater was used for the 30 ppt treatment. These salinity levels were selected to represent conditions across the freshwater-to-coastal-water gradient relevant to the early-life migration of *E. j. sinensis*. The 0 ppt treatment represented the freshwater environment used for juvenile grow-out, 15 ppt represented an intermediate brackish-water condition that may be encountered during the seawater-to-freshwater transition, and 30 ppt represented a high-salinity condition near the upper end of the salinity range reported for the Yangtze River estuary. The 30 ppt treatment also corresponded to the treated coastal seawater available at the experimental facility and was included as a high-salinity boundary condition rather than as a typical long-term habitat for juvenile crabs [13,14]. Target salinities were established before stocking. Salinity was measured daily, whereas dissolved oxygen and pH were measured weekly using a salinity meter, dissolved oxygen meter, and pH meter, respectively (YHT100B, YHT840, and YHT612; Shenzhen Yuanhengtong Technology Co., Ltd., Shenzhen, China). Dissolved oxygen remained above 5.0 mg L^−1^, and pH ranged from 7.0 to 8.0. All tanks were continuously aerated, maintained under the natural photoperiod, and kept at a water depth above 50 cm. Approximately 50% of the water was replaced weekly with water preadjusted to the corresponding treatment salinity.

All groups were fed the same commercial diet purchased from Yantai Dale Feed Co., Ltd. (Yantai, China), with its composition and pellet size provided in Table S1. Crabs were fed once daily at 16:00 at an initial rate of 5% of the estimated tank biomass. Once monthly, all crabs were temporarily captured and weighed to update the biomass used for ration adjustment, and accumulated feces and uneaten feed were removed from the tank bottom. The daily ration was further adjusted according to the amount of uneaten feed observed. Mortality was checked daily, and dead crabs were removed promptly. Nine honeycomb-shaped PVC shelters (1.0 m × 0.20 m × 0.60 m) were placed in each tank.

At the end of the rearing period, crabs were harvested separately from each tank and anesthetized on ice. Three crabs were randomly selected from each tank, giving a total of nine sampled crabs per salinity treatment. Hepatopancreatic tissues from these crabs were divided for the different analyses. For histopathological and TUNEL analyses, tissue pieces from the three crabs within each tank were fixed together in 4% paraformaldehyde at 4 °C for 24 h and treated as one tank-level sample. For the biochemical, transcriptomic, metabolomic, and qRT-PCR analyses, equal wet masses of hepatopancreatic tissue from the three crabs within each tank were pooled to generate one representative sample for that tank. Each pooled sample was divided into separate aliquots for the different analyses. Equal volumes of hemolymph collected from the same three crabs within each tank were also pooled to generate one tank-level sample for serum biochemical analysis. Thus, each treatment comprised three independent tank-level pooled biological replicates (*n* = 3), with each replicate corresponding to one independent tank and consisting of samples from three crabs. The hepatopancreatic aliquots designated for biochemical and molecular analyses were immediately frozen in liquid nitrogen and stored at −80 °C until analysis.

For intestinal microbiota analysis, five cross-tank composite samples were prepared for each salinity treatment. Each composite sample contained equal amounts of intestinal content from three unique crabs, with one crab selected from each of the three replicate tanks. No crab was included in more than one composite sample; thus, 15 crabs contributed to the microbiota analysis in each treatment. This design ensured equal representation of the three tanks in every composite sample and was intended to reduce variation among individual crabs while preventing any single tank from disproportionately influencing the treatment-level microbial profile. Although the composite samples were prepared from non-overlapping sets of crabs, each included material from all three tanks; consequently, tank identity was not retained. The composite samples were therefore used to characterize treatment-level microbial profiles and were not regarded as independent tank-level experimental replicates. Samples were immediately snap-frozen in liquid nitrogen and stored at −80 °C until DNA extraction.

### 2.3. Growth Performance and Yield

At the end of the culture period, all crabs were harvested from each cement pool. Individual crabs were cleaned, blotted dry, and placed on ice for anesthesia. Measure body length, width, height and weight. Body length, body width, and body height were measured using a digital vernier caliper, as illustrated in Figure S1. Body length was defined as the distance from the median notch between the inner frontal teeth to the posterior margin of the carapace. Body width was measured as the maximum transverse distance between the outermost lateral margins of the carapace. Body height was measured as the maximum dorsoventral distance from the dorsal surface of the carapace to the ventral surface of the folded abdomen. Use the following formula to obtain the growth performance of crabs:

Survival rate (%) = nt/n0 × 100(1)

Weight gain rate (%) = (mt − m0)/m0 × 100(2)

Specific growth rate (%) = (lnmt − lnm0)/t × 100 (3)

Total production (g/m^2^) = W/S (4)

where n0 is the estimated initial number of megalopae stocked in each tank, calculated from the initial stocking biomass (40 g) and the supplier-reported mean individual weight (0.0065 g), corresponding to approximately 6154 individuals per tank; nt is the number of surviving crabs harvested from each tank; m0 is the supplier-reported mean initial body weight (0.0065 g); mt is the mean final body weight of all surviving crabs in each tank; t is the duration of the rearing period in days; W is the total harvested biomass, defined as the combined body weight of all surviving crabs harvested from each tank (g); and S is the bottom area of each tank (6 m × 4 m = 24 m^2^). All growth and production variables were calculated separately for each tank, with the three tanks per salinity treatment serving as independent experimental units (*n* = 3).

### 2.4. Histopathological Analysis of the Hepatopancreas

The tank-level hepatopancreatic samples described in Section 2.2 were fixed in 4% paraformaldehyde at 4 °C for 24 h. After fixation, the samples were dehydrated through a graded ethanol series, embedded in paraffin, and sectioned at a thickness of 5 μm. The sections were stained with hematoxylin and eosin (H&E) as previously described [37] and examined using a Nikon light microscope equipped with a U-III multipoint sensor system (Nikon, Tokyo, Japan).

Apoptotic signals were detected using a TUNEL assay as previously described [38]. Briefly, paraffin sections were deparaffinized in two changes of xylene, rehydrated through a graded ethanol series, and washed three times with PBS. The sections were permeabilized with 1% Triton X-100, treated with proteinase K, and incubated with the TUNEL reaction mixture according to the manufacturer’s instructions. After counterstaining with DAPI, the sections were washed with PBS and mounted using an antifade mounting medium. Fluorescence images were captured using an IX71 inverted fluorescence microscope (Olympus, Tokyo, Japan). Nuclei were visualized by blue DAPI fluorescence, whereas TUNEL-positive signals were detected as green fluorescence.

### 2.5. Biochemical Analysis

Hepatopancreatic tissues and hemolymph were collected to evaluate hepatopancreatic functional status, antioxidant capacity, non-specific immune responses, and digestive enzyme activities. For each salinity treatment, three tank-level pooled biological replicates were prepared. For each replicate, hepatopancreatic tissues from three randomly selected crabs within the same tank were pooled in equal amounts. The pooled hepatopancreatic samples were homogenized with ice-cold physiological saline at a ratio of 1:9 (*w*/*v*). The homogenates were centrifuged at 3500 rpm for 10 min at 4 °C, and the supernatants were collected for biochemical assays.

Antioxidant-related indicators included superoxide dismutase (SOD), catalase (CAT), glutathione peroxidase (GSH-Px), total antioxidant capacity (T-AOC), and malondialdehyde (MDA). SOD catalyzes the conversion of superoxide anions into hydrogen peroxide, whereas CAT decomposes hydrogen peroxide and GSH-Px reduces hydrogen peroxide and lipid hydroperoxides. T-AOC was used to characterize overall antioxidant capacity, and MDA was measured as an indicator of lipid peroxidation.

The nonspecific immune-related enzymes included acid phosphatase (ACP), alkaline phosphatase (AKP), and lysozyme (LZM). ACP is primarily associated with lysosomal hydrolysis and intracellular degradation, AKP participates in dephosphorylation and phosphate metabolism, and LZM contributes to antibacterial defense through the hydrolysis of bacterial peptidoglycan. Digestive enzyme activities included lipase (LPS), trypsin, pepsin, and amylase (AMS). LPS participates in lipid hydrolysis, trypsin and pepsin are involved in protein digestion, and AMS hydrolyzes starch and related carbohydrates. Total protein (TP) concentration in hepatopancreatic homogenates was determined using the BCA method and used for normalization where appropriate.

For each tank-level biological replicate, hemolymph collected from three crabs was pooled in equal volumes. Hemolymph was collected from the arthrodial membrane at the base of the third pereiopod using sterile syringes. The pooled hemolymph was maintained at 4 °C for 24 h and then centrifuged at 4000 rpm for 10 min to obtain serum. Serum alanine aminotransferase (ALT), aspartate aminotransferase (AST), and albumin (Alb) were measured as biochemical indicators associated with tissue condition. ALT and AST are aminotransferases involved in amino acid metabolism, and increased serum activities may accompany the release of intracellular enzymes following tissue injury. Alb was used as a serum protein-related biochemical indicator.

All indicators were measured using commercial assay kits supplied by Nanjing Jiancheng Bioengineering Institute (Nanjing, China), according to the manufacturer’s instructions. The catalog numbers of the kits are provided in Table S2. Absorbance or fluorescence signals were recorded using a microplate reader (SpectraMax M5, Molecular Devices, San Jose, CA, USA) and a spectrophotometer (PHILes, Nanjing, China).

### 2.6. Transcriptome and Metabolome Analysis of the Hepatopancreas

#### 2.6.1. RNA Extraction and Transcriptome Analysis

Total RNA was extracted from each tank-level pooled hepatopancreatic sample. RNA concentration and purity were assessed using a NanoDrop 2000 spectrophotometer (Thermo Fisher Scientific, Waltham, MA, USA), and RNA integrity was evaluated using an Agilent 2100 Bioanalyzer (Agilent Technologies, Santa Clara, CA, USA) or LabChip GX system (PerkinElmer, Hopkinton, MA, USA). Library preparation and RNA sequencing were performed by Biomarker Technologies Co., Ltd. (Beijing, China).

Raw reads were filtered using fastp v0.18.0 to remove adapters and low-quality sequences [39]. Clean reads were mapped to the *E. j. sinensis* reference genome (ASM2467909v1) using HISAT2 v2.2.1 [40]. Gene expression levels were calculated as fragments per kilobase of transcript per million mapped fragments (FPKM). Principal component analysis (PCA) was used to assess sample clustering. Differentially expressed genes (DEGs) among salinity treatments were identified using DESeq2 v1.40.2, with a false discovery rate (FDR) < 0.01 and an absolute log2 fold change (|log2FC|) ≥ 1 as the screening thresholds, corresponding to a fold change ≥ 2 for upregulated genes or ≤0.5 for downregulated genes. Gene Ontology (GO) and Kyoto Encyclopedia of Genes and Genomes (KEGG) enrichment analyses were then performed to identify biological processes and pathways associated with salinity exposure [41]. KEGG enrichment was evaluated using raw *p*-values without multiple-testing correction, with *p* < 0.05 used as the enrichment threshold.

#### 2.6.2. Untargeted Metabolome Analysis

Hepatopancreatic samples were extracted using methanol/acetonitrile/water (2:2:1, *v*/*v*/*v*). The extracts were sonicated in an ice-water bath for 10 min and maintained at −20 °C for 1 h. After centrifugation at 12,000 rpm for 15 min at 4 °C, 500 μL of each supernatant was collected and dried under vacuum. The dried extracts were reconstituted in 160 μL of 50% acetonitrile and centrifuged again under the same conditions. The resulting supernatants were used for liquid chromatography-mass spectrometry (LC-MS) analysis.

Metabolomic profiling was performed using a Waters Acquity I-Class PLUS ultra-performance liquid chromatography system coupled to a Waters Xevo G2-XS QTOF high-resolution mass spectrometer (Waters Corporation, Milford, MA, USA) in both positive and negative ion modes [42]. Raw data were processed using MassLynx v4.2 and Progenesis QI for peak detection, alignment, and normalization. Metabolites were annotated using the Human Metabolome Database and METLIN database. PCA and Pearson correlation analysis were used to evaluate sample reproducibility and quality-control performance. Orthogonal partial least-squares discriminant analysis (OPLS-DA) was performed using the R package ropls. Model performance was evaluated using R^2^X, R^2^Y, and Q^2^Y, which represent the explained variation in the predictor variables, the explained variation in group membership, and the predictive capability of the model, respectively. Differentially accumulated metabolites (DAMs) were screened based on variable importance in projection (VIP) > 1.0 and *p*-value < 0.05. KEGG pathway enrichment analysis of the DAMs was performed using a hypergeometric test. Enrichment was evaluated using raw *p*-values without multiple-testing correction, with *p* < 0.05 used as the enrichment threshold. Pathway ranking in the integrated KEGG analysis was also based on raw enrichment *p*-values. DEGs and DAMs were subsequently mapped to KEGG metabolic pathways, and salinity-associated transcriptomic and metabolomic changes were visualized using iPath 3.0.

### 2.7. Intestinal Bacterial Community Analysis

Total genomic DNA was extracted from each of the 15 intestinal-content composite samples described in Section 2.2 using the TGuide S96 Magnetic Soil/Stool DNA Kit (Tiangen Biotech, Beijing, China), following the manufacturer’s instructions. The V3-V4 hypervariable region of the bacterial 16S rRNA gene was amplified using primers 338F (5′-ACTCCTACGGGAGGCAGCA-3′) and 806R (5′-GGACTACHVGGGTWTCTAAT-3′). PCR products were checked by agarose gel electrophoresis and purified using the Omega DNA Purification Kit (Omega Bio-tek, Norcross, GA, USA). Purified amplicons were pooled in appropriate proportions and sequenced on the Illumina NovaSeq 6000 platform (Illumina, Inc., San Diego, CA, USA) using a paired-end 2 × 250 bp strategy.

High-quality sequences with ≥97% similarity were clustered into operational taxonomic units (OTUs) using USEARCH v10.0 [43]. Taxonomic annotation of OTUs was performed in QIIME2 using a Naive Bayes classifier against the SILVA database (release 138.1), with a confidence threshold of 70% [44]. Alpha-diversity indices were calculated in QIIME2 to evaluate microbial richness and diversity [45]. Beta-diversity patterns were assessed using principal coordinate analysis (PCoA) and non-metric multidimensional scaling (NMDS). Hierarchical clustering was used to explore treatment-associated community patterns. RDA was performed with salinity as the sole explanatory variable to visualize its association with the bacterial community profiles; no other environmental variables were included in the ordination. Linear discriminant analysis effect size (LEfSe) was used as an exploratory approach to screen taxa contributing to differences among the treatment-level composite profiles [46].

### 2.8. Quantitative Real-Time PCR (qRT-PCR)

Quantitative real-time PCR (qRT-PCR) was performed to validate the expression patterns of selected DEGs identified by RNA-seq. Hepatopancreatic RNA samples were prepared using the same tank-level pooled biological replicates described in Section 2.2, with three replicates per salinity treatment. Total RNA was extracted using the TRIzol method. RNA concentration was measured using a NanoDrop 2000 spectrophotometer, and RNA integrity was checked by 1% agarose gel electrophoresis. Qualified RNA was reverse-transcribed into cDNA using the HisyGo RT Red SuperMix for qPCR kit (RT101-01, Vazyme, China). qRT-PCR reactions were performed using ChamQ Universal SYBR qPCR Master Mix (Q412-02, Vazyme, Nanjing, China) on a LightCycler 96 real-time PCR system (Roche Diagnostics, Rotkreuz, Switzerland). *β-actin* was used as the internal reference gene. Primers were designed using Primer Premier 5.0 and are listed in Table S3. Relative gene expression levels were calculated using the 2^−ΔΔCt^ method [47].

### 2.9. Statistical Analysis

For growth performance, production, histopathological, biochemical, and multi-omics (transcriptomic and metabolomic) data, the independent cement pool was considered the experimental unit. For intestinal microbiota analysis, five cross-tank composite samples were analyzed per salinity treatment (see Section 2.2). Although these samples were prepared from non-overlapping sets of crabs, each composite contained material from all three replicate tanks and was therefore not regarded as an independent tank-level experimental replicate. The microbiota data were used to characterize treatment-associated community profiles and were analyzed as described in Section 2.7. Tank-level quantitative data are presented as the mean ± standard error (SE). Prior to parametric analysis, data normality was assessed using the Shapiro–Wilk test, and homogeneity of variances was verified using Levene’s test. When these assumptions were met, differences among salinity treatments were analyzed by one-way analysis of variance (ANOVA), followed by Tukey’s honestly significant difference (HSD) test for multiple comparisons. When data violated the assumptions of normality or homogeneity of variance, the Kruskal–Wallis test followed by Dunn’s multiple-comparison test was applied. Statistical significance was set at *p* < 0.05. In figures, different lowercase letters indicate significant differences among treatments. All statistical analyses were performed using GraphPad Prism 7.0 and SPSS (version 20.0).

## 3. Results

### 3.1. Effects of Long-Term Salinity Exposure on Growth and Survival

**Figure 1:**
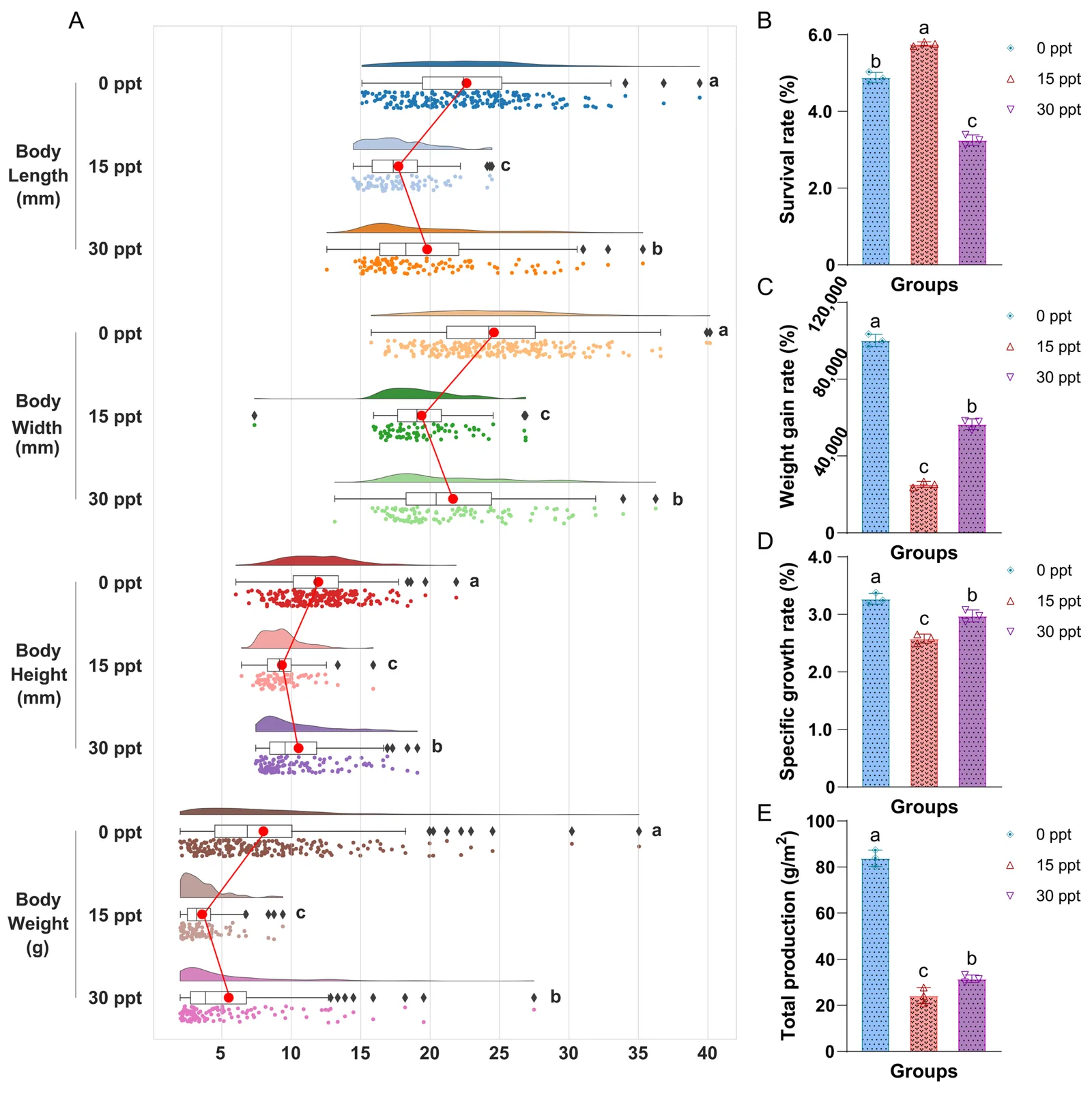
Effects of long-term rearing at different salinities on the growth, survival, and production of juvenile *E. j. sinensis*. (**A**). Final body length, body width, body height, and body weight. (**B**). Survival rate. (**C**). Weight gain rate. (**D**). Specific growth rate. (**E**). Total production. Colored points in panel (**A**) represent measurements from individual crabs. Statistical analyses of these measurements were performed using tank-level means, with each independent tank treated as one experimental unit (*n* = 3 tanks per treatment). In panels (**B**–**E**), each overlaid point represents one independent tank. Black diamonds in panel A denote observations outside the boxplot whiskers, defined as values below Q_1_ − 1.5 × IQR or above Q_3_ + 1.5 × IQR. These observations were retained in the calculation of tank-level means and were not excluded from the statistical analyses. For each measured variable, treatment groups labeled with different lowercase letters differ significantly (*p* < 0.05), whereas groups sharing the same letter do not differ significantly.

A representative juvenile *E. j. sinensis* at the end of the seven-month rearing period is shown in Figure S1A. After seven months of culture, growth performance, total production, and survival differed significantly among the three salinity treatments (Figure 1). Final body length, body width, body height, and body weight were highest at 0 ppt, intermediate at 30 ppt, and lowest at 15 ppt, with significant differences among all three treatments (Figure 1A). WGR, SGR, and total production followed the same pattern, decreasing in the order of 0 ppt, 30 ppt, and 15 ppt (Figure 1C–E). In contrast, survival was highest at 15 ppt, followed by 0 ppt and 30 ppt, with significant differences among all three treatments (Figure 1B). Thus, growth-related measurements and survival showed contrasting salinity-dependent patterns, particularly between the two saline treatments: crabs surviving at 30 ppt exhibited greater growth than those at 15 ppt, whereas survival was higher at 15 ppt.

### 3.2. Salinity-Associated Hepatopancreatic Injury and Apoptotic Signals

**Figure 2:**
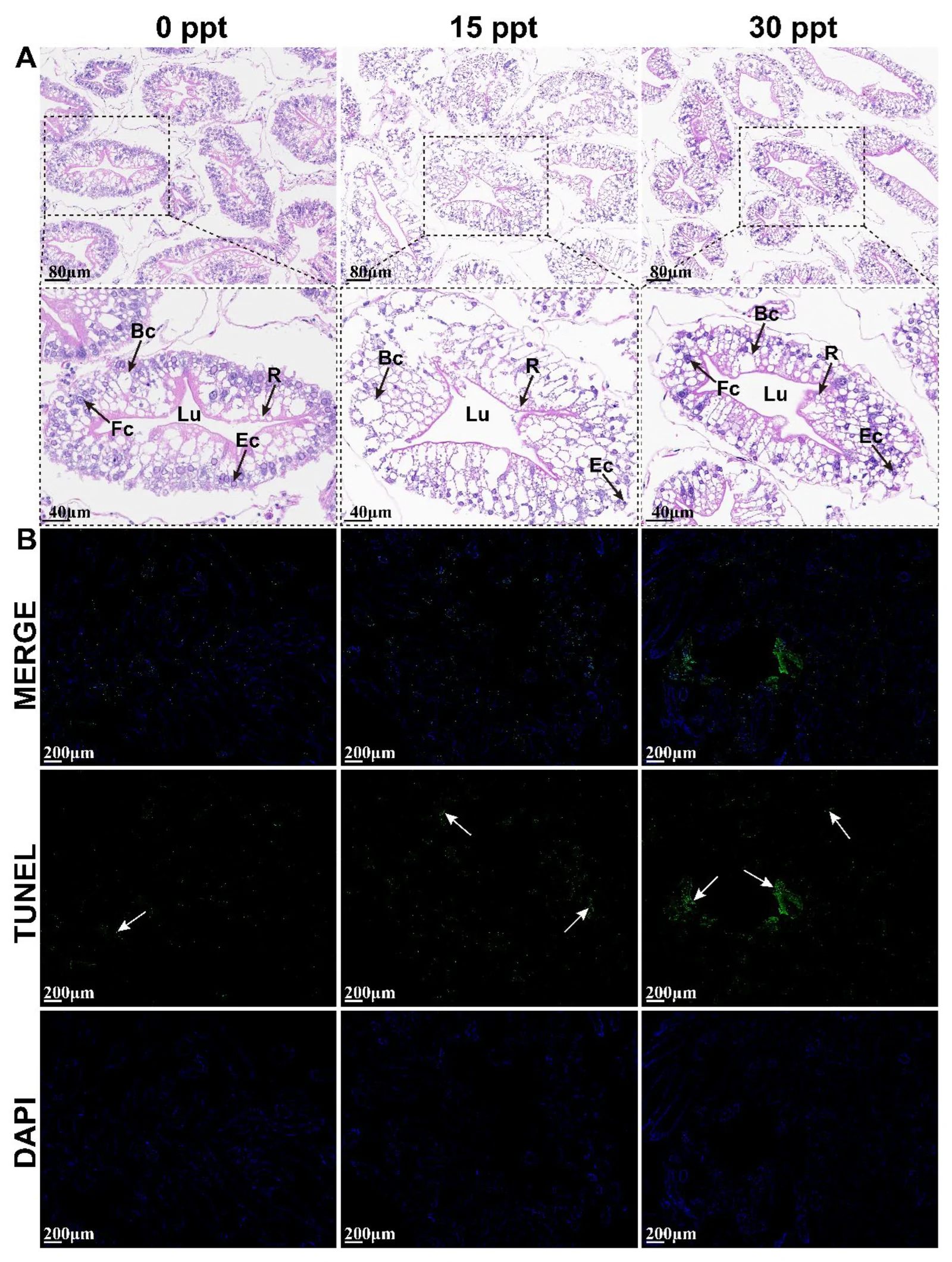
Hepatopancreatic histology and TUNEL staining of juvenile *E. j. sinensis* after long-term rearing at different salinities. (**A**). Representative hematoxylin and eosin-stained sections of the hepatopancreas. Lu, lumen; Bc, blister-like cell; Fc, fibrillar cell; Rc, resorptive cell; Ec, embryonic cell. (**B**). Representative fluorescence images of TUNEL-stained hepatopancreatic sections. TUNEL-positive signals are shown in green, and nuclei counterstained with DAPI are shown in blue. White arrows indicate representative TUNEL-positive signals.

Histological examination revealed marked salinity-associated alterations in the hepatopancreas of juvenile crabs (Figure 2A). In the 0 ppt group, hepatopancreatic tubules showed a relatively intact structure, with clear epithelial boundaries, compact tubular organization, and narrow lumens. In contrast, crabs exposed to 15 ppt displayed overt tissue injury, including enlarged lumens, epithelial vacuolation, partial detachment of the basement membrane, and local exfoliation of epithelial cells. These pathological changes were further exacerbated in the 30 ppt group, which exhibited severe tubular disarray, extensive cytoplasmic vacuolation, marked epithelial exfoliation, and pronounced basement membrane disruption (Figure 2A).

TUNEL staining showed few positive signals in hepatopancreatic sections from the 0 ppt group (Figure 2B). More extensive TUNEL-positive staining was observed in the 15 and 30 ppt groups, with the strongest signals occurring at 30 ppt. Overall, both hepatopancreatic structural alterations and TUNEL-positive staining became more evident as salinity increased.

### 3.3. Biochemical Responses to Long-Term Salinity Exposure

**Figure 3:**
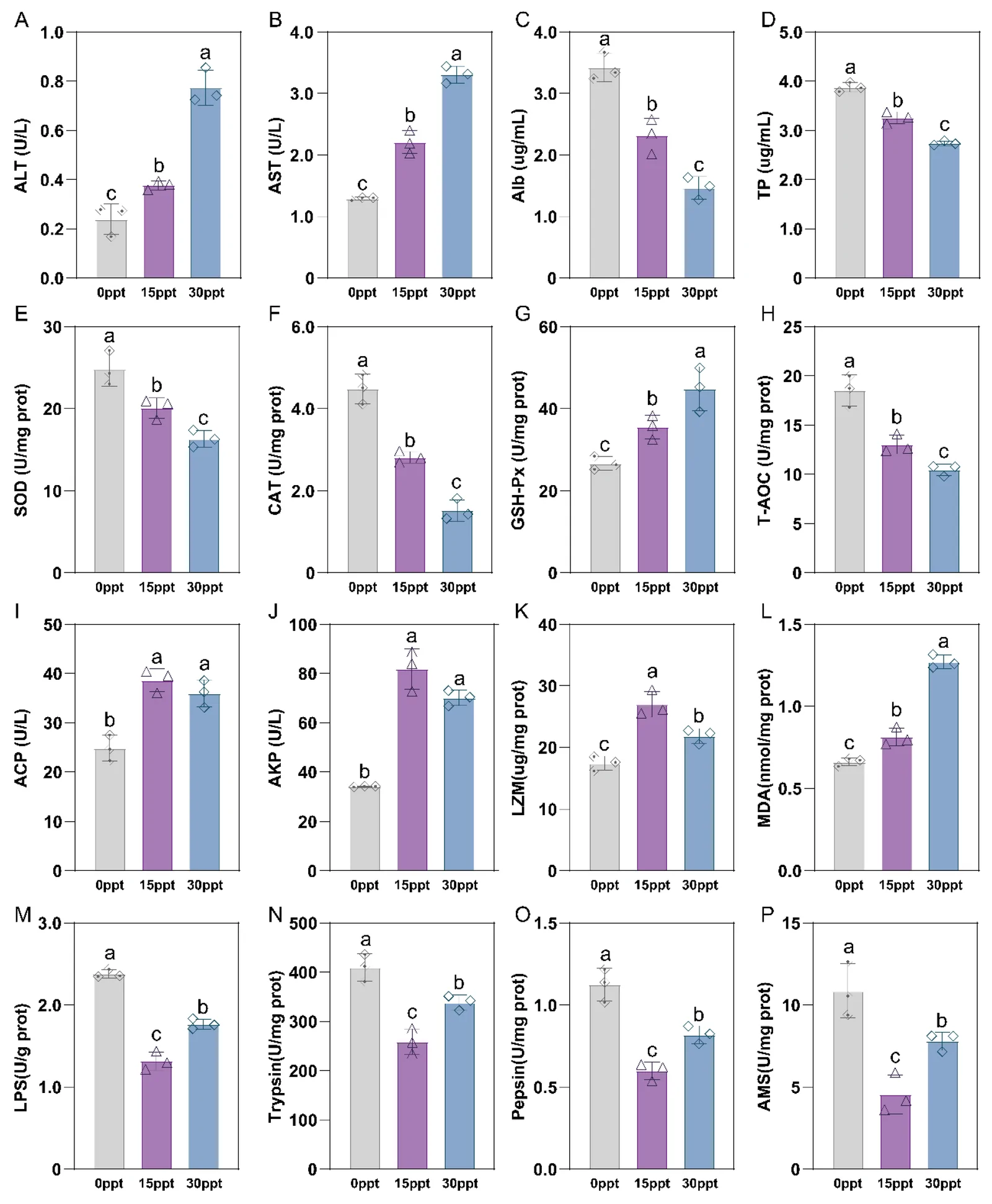
Effects of long-term rearing at different salinities on serum and hepatopancreatic biochemical indicators in juvenile *E. j. sinensis*. Serum alanine aminotransferase (ALT; (**A**)), aspartate aminotransferase (AST; (**B**)), and albumin (Alb; (**C**)); hepatopancreatic total protein (TP; (**D**)), superoxide dismutase (SOD; (**E**)), catalase (CAT; (**F**)), glutathione peroxidase (GSH-Px; (**G**)), total antioxidant capacity (T-AOC; (**H**)), acid phosphatase (ACP; (**I**)), alkaline phosphatase (AKP; (**J**)), lysozyme (LZM; (**K**)), malondialdehyde (MDA; (**L**)), lipase (LPS; (**M**)), trypsin (**N**), pepsin (**O**), and amylase (AMS; (**P**)). ALT and AST represent aminotransferase activities; Alb and TP reflect serum- and tissue-protein-related biochemical status, respectively; SOD, CAT, GSH-Px, T-AOC, and MDA characterize antioxidant and lipid-peroxidation status; ACP, AKP, and LZM represent nonspecific immune-related enzymatic responses; and LPS, trypsin, pepsin, and AMS characterize digestive capacity. For each biochemical indicator, treatment groups labeled with different lowercase letters differ significantly (*p* < 0.05), whereas groups sharing the same letter do not differ significantly. Statistical comparisons were based on three independent tanks per treatment (*n* = 3).

Serum ALT and AST activities increased progressively from 0 to 30 ppt, whereas serum Alb concentration decreased; significant differences were detected among all three treatments (Figure 3A–C). The TP concentration in the hepatopancreatic homogenate also decreased progressively with increasing salinity, with significant differences among the three treatments (Figure 3D).

SOD and CAT activities and T-AOC decreased progressively from 0 to 30 ppt, whereas GSH-Px activity and MDA content increased. For each indicator, significant differences were detected among all three treatments (Figure 3E–H,L).

ACP and AKP activities were significantly higher in the 15 and 30 ppt groups than in the 0 ppt group, with no significant difference between the two saline treatments (Figure 3I,J). The LZM level was highest at 15 ppt, intermediate at 30 ppt, and lowest at 0 ppt, with significant differences among all three treatments (Figure 3K). LPS, trypsin, pepsin, and AMS activities were highest at 0 ppt, intermediate at 30 ppt, and lowest at 15 ppt; significant differences were detected among all three treatments for each enzyme (Figure 3M–P).

### 3.4. Hepatopancreatic Transcriptomic Responses to Salinity

**Figure 4:**
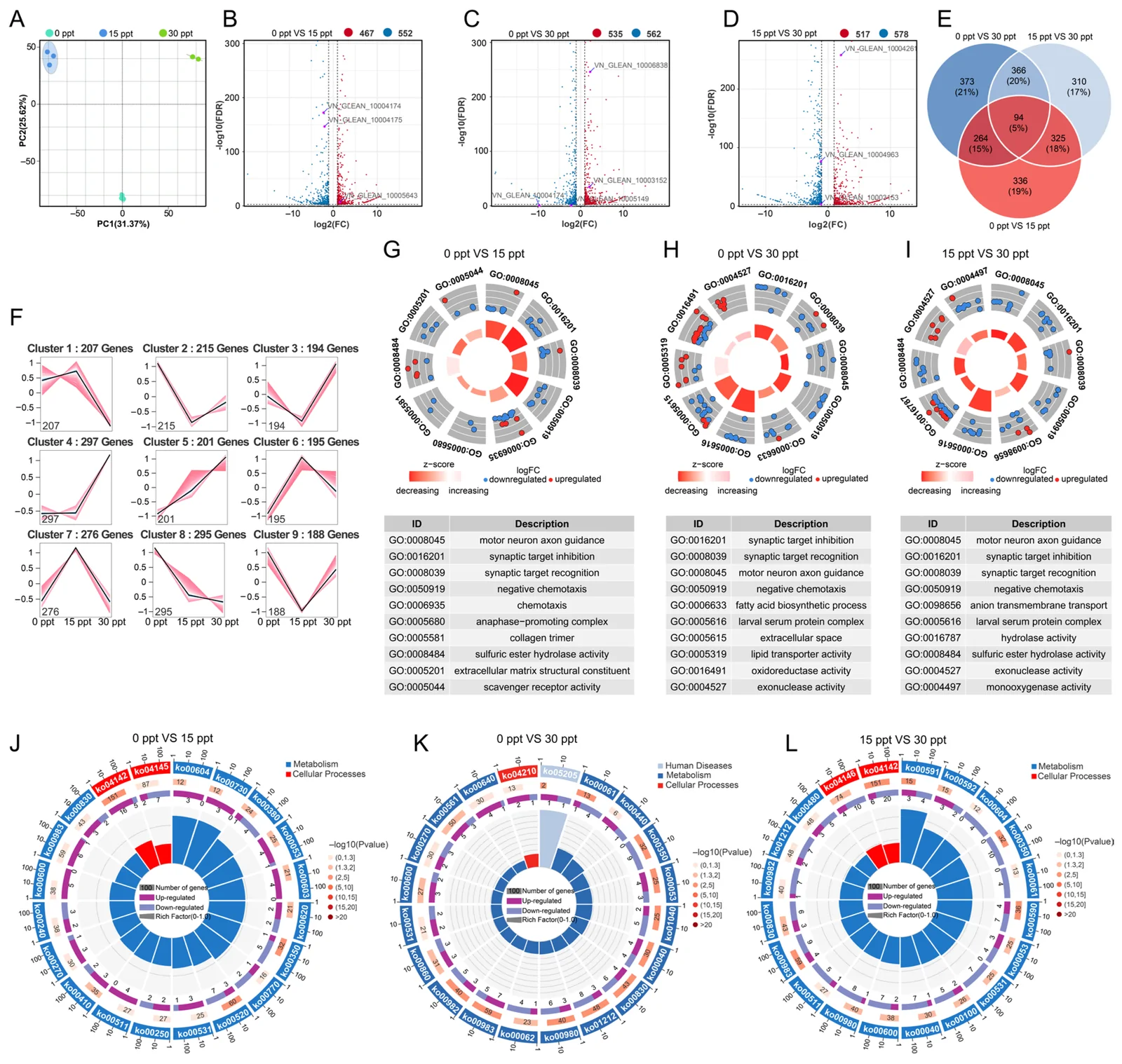
Hepatopancreatic transcriptomic profiles of juvenile *E. j. sinensis* reared at different salinities. (**A**). principal component analysis (PCA). Volcano plot of significant DEGs of 0 ppt vs. 15 ppt (**B**), 0 ppt vs. 30 ppt (**C**), and 15 ppt vs. 30 ppt (**D**). Red dots, upregulated; blue dots, downregulated. (**E**). Venn diagram. (**F**). Modular mapping based on variation trend of DEGs. The GO and KEGG enrichment analyses of DEGs in 0 ppt vs. 15 ppt (**G**,**J**), 0 ppt vs. 30 ppt (**H**,**K**), and 15 ppt vs. 30 ppt (**I**,**L**) comparisons. KEGG enrichment in panels J–L was evaluated using raw *p*-values without multiple-testing correction.

RNA sequencing of nine hepatopancreatic samples generated 57.73 Gb of clean data. Each sample yielded at least 5.87 Gb of clean data, with Q30 values of at least 93.28% (Table S4). PCA showed clear separation among the three salinity treatments, while the biological replicates within each treatment clustered together (Figure 4A).

Differential expression analysis identified 1019, 1097, and 1095 DEGs in the 0 ppt vs. 15 ppt, 0 ppt vs. 30 ppt, and 15 ppt vs. 30 ppt comparisons, respectively (Figure 4B–D; Table S5). Among these, 467/552, 535/562, and 517/578 genes were upregulated/downregulated in the 0 ppt vs. 15 ppt, 0 ppt vs. 30 ppt, and 15 ppt vs. 30 ppt comparisons, respectively. Venn analysis showed that 94 DEGs were shared across all three comparisons (Figure 4E). Mfuzz analysis grouped the 2068 DEGs into nine clusters, with clusters 2 and 9 broadly mirroring the treatment patterns of growth-related measurements and total production (Figure 4F).

**Figure 5:**
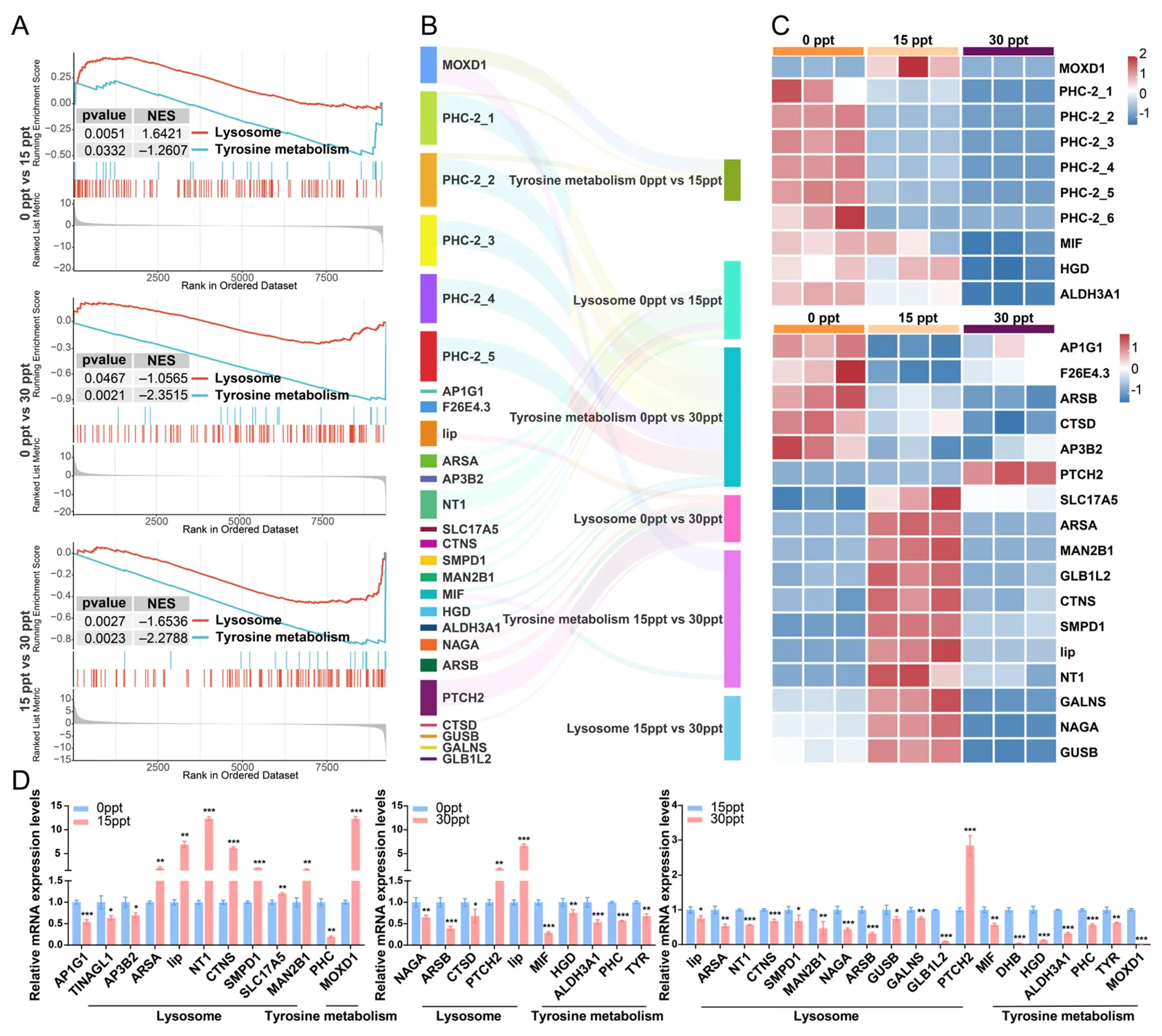
Long-term culture at different salinity significantly altered Lysosome and Tyrosine metabolism signaling pathway of 0 ppt vs. 15 ppt, 0 ppt vs. 30 ppt, and 15 ppt vs. 30 ppt. (**A**). GSEA analysis. (**B**). Sankey diagrams showed that in all three comparisons, Lysosome and Tyrosine metabolism and related DEGs were identified. (**C**). Heatmap depicting common genes from the Lysosome and Tyrosine metabolism pathway in the hepatopancreas. (**D**). qRT-PCR detection of core DEGs of the Lysosome and Tyrosine metabolism pathway in the hepatopancreas. Asterisks indicate significant differences between the indicated treatments (* *p* < 0.05, ** *p* < 0.01, and *** *p* < 0.001).

GO enrichment analysis revealed that DEGs were mainly associated with metabolic processes, cellular component organization, and stress-responsive signaling pathways, with the specific enriched terms varying among comparisons (Figure 4G–I). KEGG enrichment analysis identified several metabolic and stress-related pathways associated with salinity exposure. Notably, the Lysosome and Tyrosine metabolism pathways were identified across all three comparisons (Figure 4J–L). GSEA further revealed that the Lysosome pathway was enriched in the upregulated gene set in the 0 ppt vs. 15 ppt comparison but showed a downregulated trend in the 0 ppt vs. 30 ppt and 15 vs. 30 ppt comparisons (Figure 5A). In contrast, the Tyrosine metabolism pathway exhibited a consistent downregulated trend across all three comparisons (Figure 5A). A Sankey diagram illustrated shared pathway-gene relationships involving Lysosome and Tyrosine metabolism (Figure 5B). qRT-PCR validation of selected DEGs showed expression patterns consistent with RNA-seq data, supporting the reliability of the transcriptomic analysis (Figure 5C,D).

### 3.5. Hepatopancreatic Metabolomic Responses to Salinity

**Figure 6:**
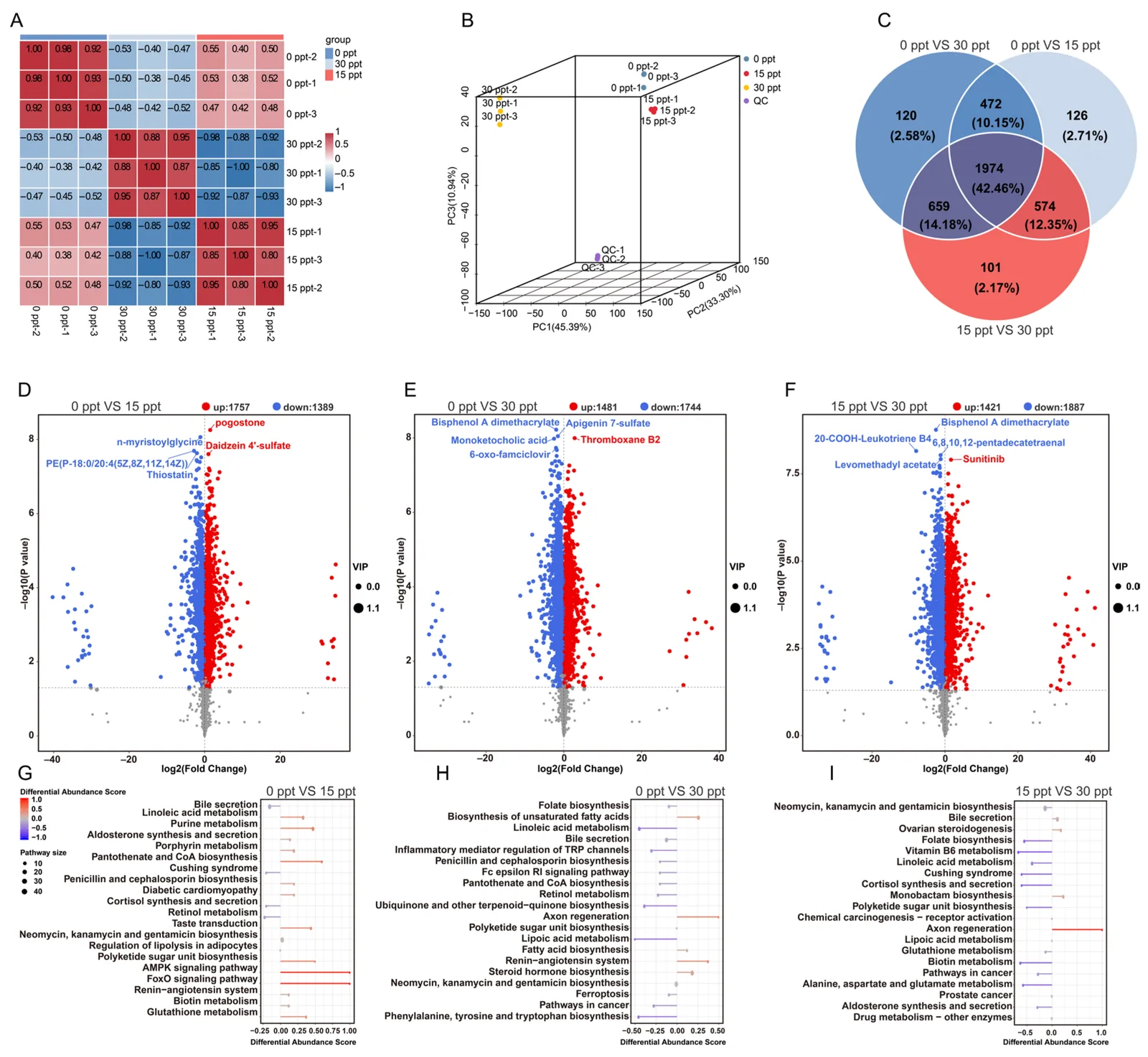
Hepatopancreatic metabolomic profiles of juvenile *E. j. sinensis* reared at different salinities. (**A**). Pearson correlation matrix of the metabolomic samples. (**B**). Principal component analysis (PCA) of the experimental and pooled quality-control (QC) samples. (**C**). Venn diagram showing shared and comparison-specific DAMs. Volcano plots of DAMs in the 0 ppt vs. 15 ppt (**D**), 0 ppt vs. 30 ppt (**E**), and 15 ppt vs. 30 ppt (**F**) comparisons. Red dots, upregulated; blue dots, downregulated; grey dots, no significant difference. KEGG pathway differential abundance score plots for the 0 ppt vs. 15 ppt (**G**), 0 ppt vs. 30 ppt (**H**), and 15 ppt vs. 30 ppt (**I**) comparisons. The x-axis represents differential abundance score, y-axis represents differential pathway name, length of segment represents absolute value of differential score, size of endpoint of segment represents number of DAMs, score of 1 represents overall upward trend of differential metabolite in pathway, score of -1 represents overall downward trend of differential metabolite in pathway. KEGG pathway enrichment was evaluated using raw *p*-values without multiple-testing correction.

Untargeted LC-QTOF metabolomics was performed to characterize hepatopancreatic metabolic responses to long-term salinity exposure. In total, 4649 metabolites were detected across nine samples, including 2665 in positive-ion mode and 1984 in negative-ion mode. Pearson correlation analysis demonstrated strong intra-group correlations, while PCA revealed distinct separation among salinity treatments (Figure 6A,B).

Analysis of differentially accumulated metabolites (DAMs) revealed broad salinity-associated metabolic remodeling with 1757/1389, 1481/1744, and 1421/1887 metabolites upregulated/downregulated in the 0 ppt vs. 15 ppt, 0 ppt vs. 30 ppt and 15 vs. 30 ppt comparisons, respectively (Figure 6D–F). Venn analysis showed that 1974 DAMs, accounting for 42.46% of all detected metabolites, were shared among the three comparisons (Figure 6C). OPLS-DA further showed separation between the salinity treatments (Figure S2). The R^2^X, R^2^Y, and Q^2^Y values were 0.895, 1.000, and 0.997 for the 0 ppt vs. 15 ppt comparison; 0.917, 1.000, and 0.997 for the 0 ppt vs. 30 ppt comparison; and 0.903, 1.000, and 0.998 for the 15 ppt vs. 30 ppt comparison, respectively. The first predictive component explained 77%, 79%, and 81% of the variation in the three comparisons, respectively. These values indicated good model fit and high apparent predictive performance within the present dataset, although they should be interpreted in light of the limited number of biological replicates. The metabolic features showing the largest positive and negative fold changes in each comparison are presented in Figure S3.

KEGG-based differential abundance scoring revealed comparison-specific pathway patterns (Figure 6G–I). DAMs mapped to the AMPK and FoxO signaling pathways showed predominantly increased abundance in the 0 ppt vs. 15 ppt comparison. DAMs associated with axon regeneration also showed a predominantly increasing pattern in the 0 ppt vs. 30 ppt and 15 ppt vs. 30 ppt comparisons. These results indicate that long-term salinity exposure was associated with extensive metabolic remodeling in the hepatopancreas, particularly in pathways related to energy sensing and stress responses.

### 3.6. Integrated Transcriptomic and Metabolomic Analysis

To examine the relationships between the transcriptomic and metabolomic responses to long-term salinity exposure, the two datasets were integrated using two-way orthogonal partial least squares (O2PLS). Gene and metabolite loading plots were generated to visualize their contributions to the joint model (Figure S4A). Based on the lengths of their loading vectors across the first two dimensions, the 15 DEGs and DAMs with the largest contributions were selected and displayed in bar plots (Figure S4B). Pearson correlation analysis was subsequently performed between DEGs and DAMs. Gene-metabolite pairs with an absolute correlation coefficient greater than 0.8 (|r| > 0.8) and a *p*-value below 0.05 were integrated with their fold-change values in nine-quadrant plots (Figure S5). DEGs and DAMs in quadrants 3 and 7 showed concordant changes, whereas those in quadrants 1 and 9 showed opposing changes.

Joint KEGG analysis was then performed for the DEGs and DAMs. The 30 pathways containing the largest numbers of DEGs and DAMs were visualized using bubble plots (Figure S6A). The ten leading pathways, ranked according to gene cluster frequency and raw enrichment *p*-values, were further examined using KEGG Markup Language (KGML) analysis to visualize the relationships among pathways, DEGs, and DAMs (Figure S6B). Among these, the Lysosome and Tyrosine metabolism pathways emerged as prominent candidates in the integrated response to long-term salinity exposure, with both DEGs and DAMs mapped to each pathway [48,49].

### 3.7. Salinity-Associated Variation in Intestinal Bacterial Communities Profiles

**Figure 7:**
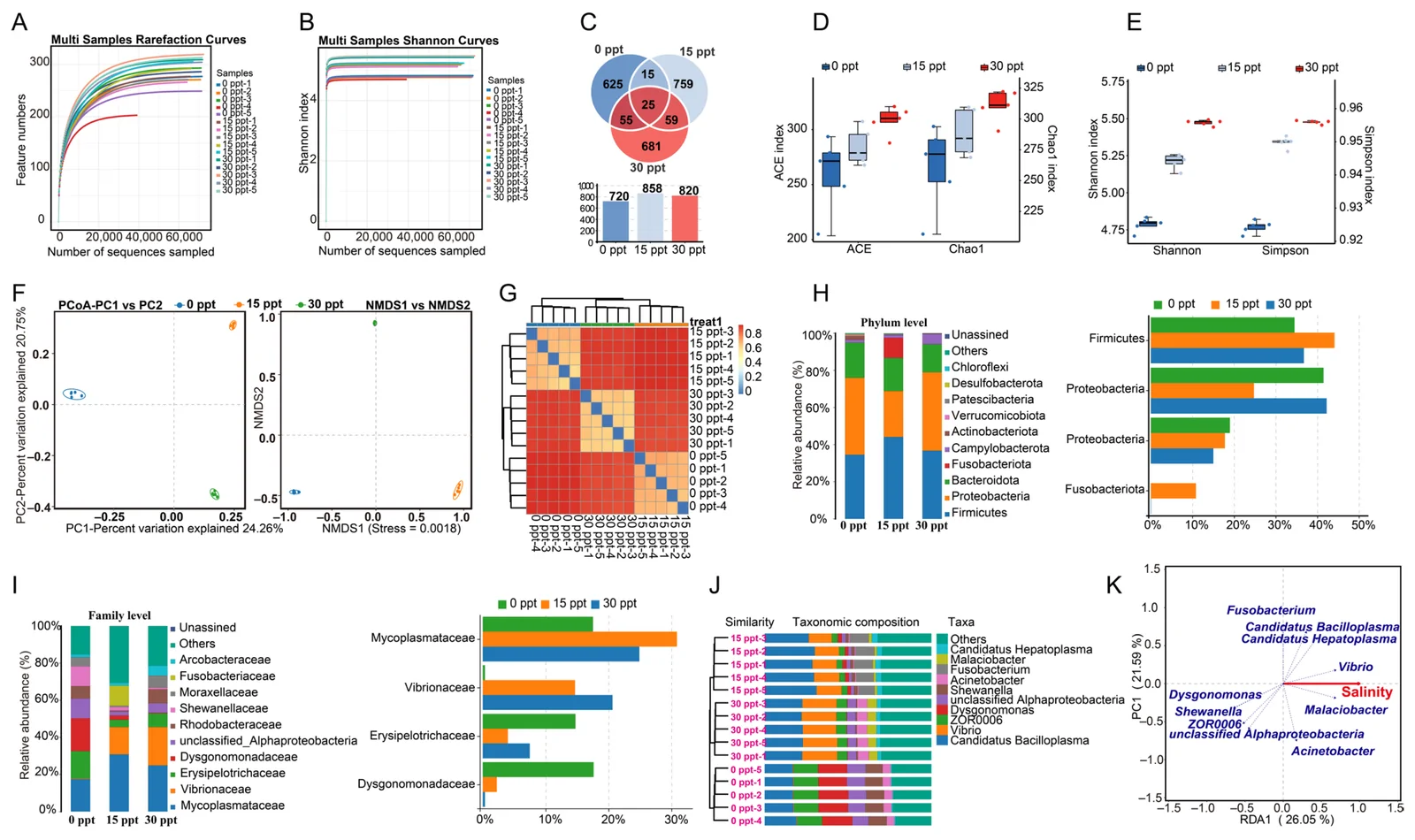
Intestinal bacterial communities of juvenile *E. j. sinensis* reared at different salinities. (**A**). Rarefaction curve. (**B**). Shannon index curve. (**C**). Venn diagram analysis of OTUs. (**D**,**E**). Alpha-diversity indices. (**F**). Principal coordinates analysis (PCoA) and non-metric multidimensional scaling (NMDS). (**G**). Sample cluster heat map analysis. (**H**,**I**) Bacterial community structure of the intestinal sample groups at the phylum and family level. (**J**). Sample hierarchical cluster tree and genus level abundance histogram. Only phylum, family and genus with top 10 abundance levels are shown. (**K**) Redundancy analysis (RDA) illustrating associations between salinity and dominant bacterial genera. Salinity was the sole explanatory variable included in the ordination. The red vector represents salinity, and the blue vectors represent bacterial genera. Vectors pointing in the same or opposite direction indicate positive or negative associations with salinity, respectively, and a smaller angle indicates a stronger association. Vector length reflects the strength of the association with the ordination pattern. Because the five cross-tank composite samples per treatment were not independent tank-level replicates, alpha-diversity and relative abundance data are presented as descriptive treatment-level comparisons, and no formal inferential statistical tests were applied to these variables.

A total of 1,163,850 paired-end reads were generated by bacterial 16S rRNA gene amplicon sequencing of the 15 intestinal-content samples. After quality control and assembly, 1,068,797 clean reads were retained, with an average of 71,253 reads per sample. Rarefaction curves for OTU richness and Shannon diversity reached a plateau at sequencing depths above 40,000 reads, indicating sufficient sequencing coverage for characterizing the bacterial communities represented in these samples (Figure 7A,B).

Venn analysis identified 2219 OTUs across all samples, with 25 OTUs shared among the three salinity treatments (Figure 7C). The numbers of treatment-specific OTUs were 625, 759, and 681 in the 0 ppt, 15 ppt, and 30 ppt groups, respectively, showing differences in OTU composition among treatments. Descriptive comparisons showed that the ACE and Chao1 indices were higher at 30 ppt than at 0 ppt, while the Shannon and Simpson indices tended to increase with salinity (Figure 7D,E). These patterns indicated greater bacterial richness and diversity in the composite samples from the higher-salinity treatments.

PCoA and NMDS showed clear separation of the bacterial community profiles among the three salinity treatments (Figure 7F). The sample-distance heatmap also showed greater similarity among composite samples from the same treatment than among those from different treatments (Figure 7G). Taxonomic profiling identified Firmicutes, Proteobacteria, Bacteroidota, and Fusobacteriota as the dominant phyla, whereas *Mycoplasmataceae*, *Vibrionaceae*, *Erysipelotrichaceae*, and *Dysgonomonadaceae* predominated at the family level (Figure 7H,I). At the genus level, *Candidatus Bacilloplasma*, *Vibrio*, *ZOR0006*, and *Dysgonomonas* were among the most abundant taxa (Figure 7J). The composite profiles from the higher-salinity treatments showed greater relative abundances of *Candidatus Bacilloplasma* and *Vibrio* and lower relative abundances of *ZOR0006* and *Dysgonomonas*.

RDA, with salinity as the sole explanatory variable, showed an association between salinity and variation in the intestinal bacterial community profiles. The salinity vector was oriented in the same direction as *Vibrio* and *Malaciobacter* and in the opposite direction to *Dysgonomonas* (Figure 7K). Exploratory LEfSe analysis identified taxa that differentiated the composite profiles among salinity treatments (Figures S7 and S8). *Vibrio* and *Vibrionaceae* characterized the 30 ppt profiles, *Mycoplasmatales* characterized the 15 ppt profiles, and *Dysgonomonas* and *Alphaproteobacteria* characterized the 0 ppt profiles. Spearman correlation network analysis further showed treatment-associated co-occurrence patterns among dominant genera, with *Candidatus Bacilloplasma* and *Vibrio* occurring as prominent taxa across all salinity treatments (Figure S9). Overall, the intestinal bacterial community profiles differed among salinity treatments, with the higher-salinity profiles showing a greater relative abundance of *Vibrio* and a lower relative abundance of *Dysgonomonas*.

## 4. Discussion

Salinity can affect crustacean growth by increasing osmoregulatory and energetic demands and by altering feeding and digestive processes [50,51,52]. In the present study, crabs reared at 0 ppt had the highest final body measurements, WGR, SGR, and total production, consistent with the freshwater growth phase of juvenile *E. j. sinensis* [10,11,12]. Both saline treatments reduced growth performance and production relative to 0 ppt. The accompanying reductions in lipase, trypsin, pepsin, and amylase activities suggest that lower digestive capacity may have contributed to this growth limitation, although enzyme activities alone do not directly measure feed intake or nutrient assimilation. Growth and survival responded differently between the two saline treatments. Crabs surviving at 30 ppt were larger and had higher digestive enzyme activities than those at 15 ppt, but the 30 ppt treatment had the lowest survival rate. The partial recovery of digestive enzyme activities at 30 ppt may represent a compensatory response to increased energetic demands rather than an improvement in physiological condition. Because growth and biochemical measurements were obtained only from surviving animals, the higher values observed at 30 ppt should not be interpreted as evidence that this salinity was favorable for the population as a whole. They may also partly reflect reduced competition at the lower survivor density, because density can influence the growth and survival of juvenile crabs [53], or the selective survival of individuals that tolerated high salinity better. Conversely, 15 ppt produced the highest survival rate but the lowest growth performance and total production. At 15 ppt, maintaining osmotic balance and other physiological functions may have left less energy available for somatic growth. However, energy allocation was not measured directly in this study. Because the physiological variables were measured only at harvest, we could not determine whether the non-linear pattern resulted from acclimation, selective survival, or differences in survivor density. It should therefore not be taken as direct evidence of physiological acclimation and is more cautiously described as a salinity-specific response. Taken together, 0 ppt supported the best overall growth and production performance, whereas 15 ppt provided a survival advantage without maximizing growth. At 30 ppt, the greater growth of the surviving individuals did not offset the markedly lower survival rate and therefore did not translate into an overall benefit to animal performance.

The hepatopancreas performs essential roles in digestion, nutrient storage, metabolism, detoxification, and immune-related functions in crustaceans, making its structural and functional integrity an important indicator of animal health [20,54]. In the present study, serum ALT and AST activities increased progressively with salinity, whereas serum Alb concentration and TP concentration in the hepatopancreatic homogenate decreased. When considered alongside the histological alterations, the elevated serum ALT and AST activities were consistent with the leakage of intracellular enzymes into the hemolymph following tissue injury [21,55]. The concurrent decreases in serum Alb and hepatopancreatic TP further indicated alterations in protein-related biochemical status under prolonged saline rearing. This interpretation was supported by the histological observations. Compared with the relatively intact hepatopancreatic tubules at 0 ppt, the saline treatments showed enlarged tubular lumens, epithelial vacuolation, epithelial sloughing, and disruption of tissue organization, with the most extensive alterations occurring at 30 ppt (Figure 2A). TUNEL-positive staining also became more prominent as salinity increased and was strongest at 30 ppt (Figure 2B). Collectively, the biochemical, histological, and TUNEL findings indicate that prolonged saline rearing adversely affected hepatopancreatic condition, with the greatest damage observed at 30 ppt. Thus, unlike the partial recovery of growth and digestive enzyme activities among the surviving crabs at 30 ppt, hepatopancreatic condition showed no comparable improvement. Future studies incorporating additional salinity treatments at 35–40 ppt and repeated measurements during rearing are needed to determine whether the partial recovery of some variables persists at higher salinities and to define the upper salinity tolerance of juvenile *E. j. sinensis*.

Redox imbalance occurred concomitantly with hepatopancreatic injury under saline conditions. Maintaining osmotic and ionic balance requires considerable energy expenditure, and sustained salinity challenge can increase metabolic demand and ROS formation [56,57,58,59]. In the present study, SOD and CAT activities and T-AOC decreased progressively with increasing salinity (Figure 3E,F,H), indicating a reduced capacity to remove superoxide radicals and hydrogen peroxide. When ROS production exceeds antioxidant capacity, ROS can attack polyunsaturated fatty acids in cellular and organelle membranes, initiating lipid peroxidation and producing reactive aldehydes such as MDA [60,61]. Accordingly, the increase in MDA observed in this study indicated greater lipid peroxidation under saline conditions [60,61,62]. Lipid peroxidation can compromise membrane integrity and mitochondrial function [61,63]. Dysfunctional mitochondria may then produce additional ROS, creating a positive-feedback cycle that further amplifies oxidative injury [64]. Oxidative damage to membrane lipids, proteins, and DNA can subsequently disrupt hepatopancreatic epithelial integrity and promote apoptosis when cellular repair capacity is exceeded [38,61,65]. This sequence provides a plausible explanation for the concurrent increase in MDA, epithelial vacuolation and sloughing, serum ALT and AST activities, and TUNEL-positive signals, particularly at 30 ppt. Although GSH-Px activity increased with salinity, this change occurred alongside declining overall antioxidant capacity and increasing lipid peroxidation and hepatopancreatic damage. Collectively, prolonged saline exposure was accompanied by reduced antioxidant capacity, increased lipid peroxidation, and hepatopancreatic injury, supporting an association among these responses.

Crustaceans rely primarily on innate defense mechanisms, in which ACP, AKP, and LZM participate in lysosomal and antimicrobial processes [66,67,68,69]. ACP contributes to lysosomal hydrolysis and the intracellular degradation of foreign or damaged material, AKP participates in dephosphorylation and phosphate-related cellular processes, and LZM hydrolyzes bacterial peptidoglycan and contributes to bacterial clearance [66,67,68,69]. In the present study, ACP and AKP activities were higher in both saline treatments than at 0 ppt, whereas LZM activity was highest at 15 ppt and decreased at 30 ppt, although it remained higher than at 0 ppt (Figure 3I–K). The simultaneous elevation of these enzymes at 15 ppt may reflect a compensatory response that supported lysosomal processing and nonspecific antibacterial defense during prolonged salinity exposure. By facilitating the degradation of foreign or damaged material and supporting bacterial clearance, these processes could help maintain cellular homeostasis and basal defense under salinity stress, providing a plausible functional link to the higher survival observed at 15 ppt. However, ACP and AKP activities also remained elevated at 30 ppt, where survival was lowest and hepatopancreatic injury was most pronounced. Therefore, the activities of these enzymes alone cannot explain the survival pattern or demonstrate an overall enhancement of immunity. Overall, these enzyme responses are best interpreted as part of a compensatory defense response associated with, but not demonstrated to cause, the survival advantage at 15 ppt.

The transcriptomic and metabolomic results provide a molecular context for the contrasting growth and survival responses observed among salinity treatments. GO enrichment included terms related to nutrient reservoir activity and storage protein complexes. Because vitellogenins serve as nutrient reserves and lipid carriers, whereas apolipoproteins participate in lipid transport, the enrichment of these terms may reflect altered nutrient storage and lipid allocation and may be related to the reduced growth observed at 15 and 30 ppt [70,71,72]. Previous research in female *E. j. sinensis* showed that salinity challenge altered the utilization of major energy substrates: carbohydrates were mobilized first to meet the increased energy demand, while proteins and lipids also contributed to energy supply and osmoregulation [59]. In the present study, reduced amylase activity suggested a lower capacity to hydrolyze dietary starch, while the decreases in lipase, trypsin, and pepsin indicated concurrent reductions in lipid and protein digestion. At 15 ppt, DAMs mapped to AMPK and FoxO signaling were predominantly increased. Because these pathways link cellular energy and redox status with carbohydrate and lipid metabolism and stress responses [22,23,24,73], this pattern is consistent with an adjustment of energy metabolism toward meeting the demands of osmoregulation and cellular maintenance. Meanwhile, enrichment of fatty acid biosynthesis, linoleic acid metabolism, and pantothenate and CoA biosynthesis, together with changes in nutrient storage and transport proteins, was consistent with remodeling of fatty acid synthesis, lipid transport, and CoA-dependent substrate metabolism [16]. These changes may have altered the balance between lipid storage and utilization, thereby reducing the nutrients available for tissue growth.

Integrated analysis further identified Lysosome and Tyrosine metabolism as prominent pathways containing both DEGs and DAMs. Tyrosine metabolism showed negative enrichment as salinity increased (Figure 5A). Because tyrosine catabolism supplies intermediates for energy metabolism, its negative enrichment may indicate reduced utilization of amino acid-derived substrates and further contribute to the metabolic constraints on growth [74,75]. In contrast, the Lysosome pathway showed positive enrichment at 15 ppt but negative enrichment in the comparisons involving 30 ppt. Positive Lysosome enrichment at 15 ppt was consistent with greater reliance on lysosome-related degradation and recycling of damaged proteins and organelles [23,24,25,76,77,78]. This cellular-maintenance response may help explain the higher survival at 15 ppt, although the low WGR, SGR, and total production indicate that it did not prevent growth reduction. At 30 ppt, negative Lysosome enrichment occurred together with more extensive hepatopancreatic damage, stronger TUNEL-positive staining, and lower survival, suggesting that lysosome-related cellular maintenance was not sustained under high salinity. Taken together, these pathway-level changes provide a plausible molecular explanation for the high survival but limited growth at 15 ppt and the greater tissue damage and mortality at 30 ppt.

Salinity markedly changed the intestinal bacterial community of juvenile *E. j. sinensis*, likely because bacterial taxa differ in their tolerance to saline conditions [13,79]. Alpha diversity increased with salinity and was highest at 30 ppt; however, this group showed lower growth than the 0 ppt group and the lowest survival rate, indicating that greater bacterial diversity did not correspond to better animal performance. The taxonomic changes provide further insight into the possible functional consequences of this community shift. *Candidatus Bacilloplasma*, a common resident of the crustacean intestine that has been associated with digestive functions [80,81,82,83], increased under saline conditions. In contrast, the relative abundance of *Dysgonomonas* decreased as salinity increased. Previous work in juvenile *E. j. sinensis* identified *Dysgonomonas* as a potential probiotic associated with the digestion of plant-rich feed [32]. Its reduction under saline conditions may therefore have decreased the microbial contribution to complex carbohydrate utilization, consistent with the lower amylase activity and reduced growth in the saline treatments. *Alphaproteobacteria* were enriched at 0 ppt, where growth and antioxidant capacity were highest. A previous study in *L. vannamei* similarly found that an increase in *Alphaproteobacteria* following yeast extract supplementation accompanied improvements in growth and antioxidant capacity [33], suggesting that members of this class may be associated with favorable nutritional and physiological conditions. In contrast to these taxa, the relative abundance of *Vibrio* increased with salinity and was highest at 30 ppt. The occurrence of *Vibrio* in saline environments is well documented [84,85], and its increase in the present study is consistent with the selection of salt-tolerant bacteria. Because this genus contains opportunistic pathogens of cultured crustaceans, its greater abundance at 30 ppt may represent an additional health risk. This increase occurred together with the lowest survival rate, higher MDA content, more extensive hepatopancreatic damage, and lower LZM than at 15 ppt. Although 16S rRNA gene sequencing could not determine whether the detected *Vibrio* belonged to pathogenic species or strains, its expansion indicates that high salinity favored a bacterial group of concern for crab health.

**Figure 8:**
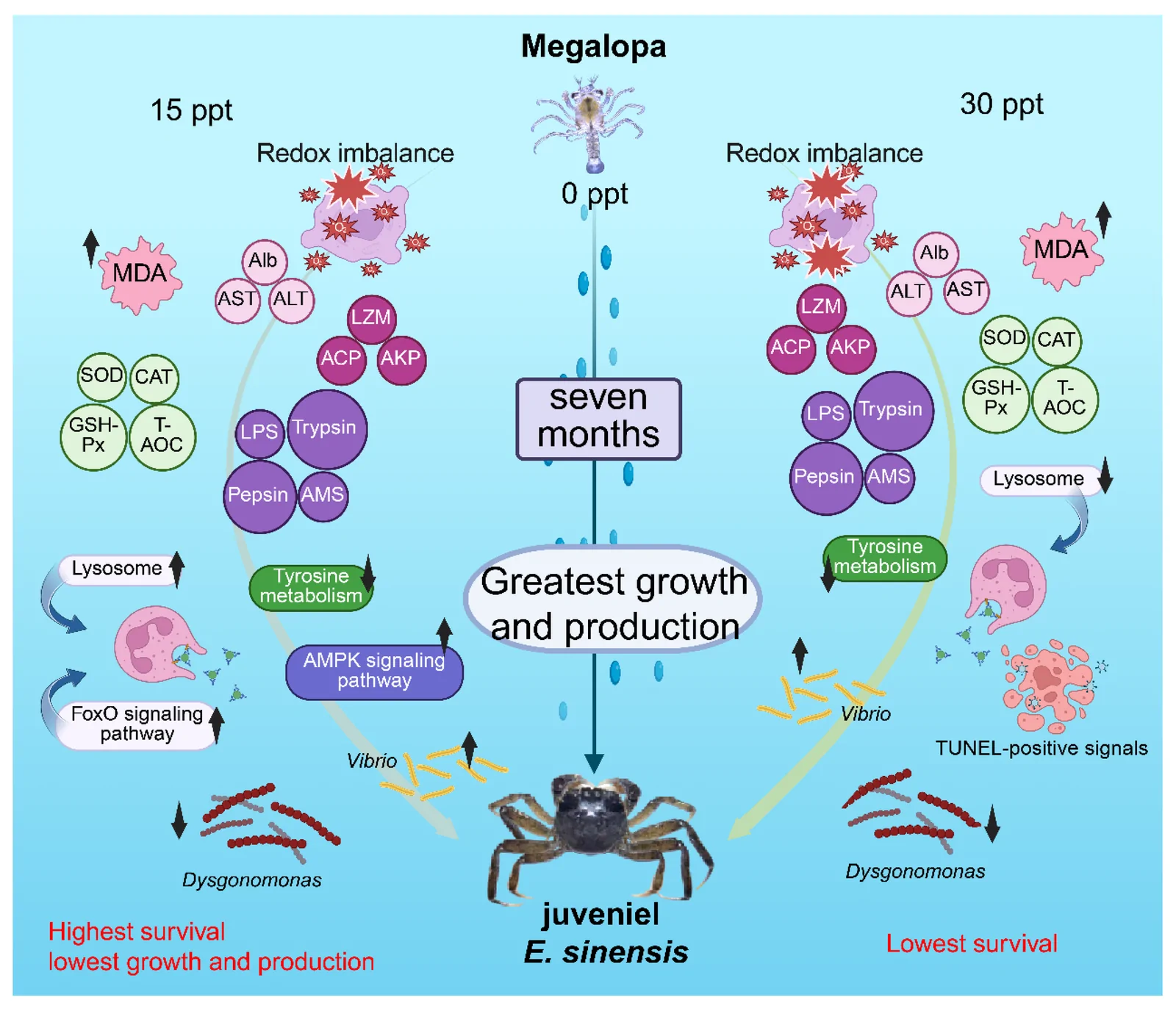
Integrated model of salinity-associated responses in juvenile *E. j. sinensis* after seven months of rearing. The 0 ppt group showed the greatest growth and production, the 15 ppt group had the highest survival but the lowest growth and production, and the 30 ppt group had the lowest survival. Relative to 0 ppt, saline rearing increased serum aminotransferase, GSH-Px, immune-related enzyme activities, and MDA, but decreased Alb, SOD, CAT, T-AOC, and digestive enzyme activities. The Lysosome pathway showed positive enrichment at 15 ppt and negative enrichment at 30 ppt, whereas Tyrosine metabolism showed negative enrichment under both saline conditions. At 15 ppt, most DAMs mapped to the AMPK and FoxO signaling pathways showed increased abundance relative to 0 ppt. TUNEL-positive signals were most pronounced at 30 ppt. *Vibrio* increased and *Dysgonomonas* decreased with salinity. Arrows indicate directions relative to 0 ppt, not effect sizes or statistical significance.

Collectively, these findings support an integrated model in which long-term salinity exposure was associated with coordinated changes in digestive capacity, redox balance, hepatopancreatic condition, innate immune-related enzyme activities, lysosome/autophagy-related pathways, and intestinal bacterial community structure (Figure 8). At 0 ppt, high digestive enzyme activities, stronger antioxidant capacity, and relatively intact hepatopancreatic structure provided physiological conditions favorable for juvenile growth. At 15 ppt, digestive enzyme activities and growth declined markedly, whereas survival was highest. The higher survival coincided with elevated ACP, AKP, and LZM activities, positive enrichment of the Lysosome pathway, and increased abundances of metabolites mapped to AMPK and FoxO signaling. Together, these changes suggest a compensatory response involving lysosomal processing, cellular recycling, and nonspecific defense, which may have helped the crabs tolerate moderate salinity despite reduced growth; however, they do not demonstrate a general enhancement of immunity or establish a causal explanation for the higher survival. At 30 ppt, however, lower SOD, CAT, and T-AOC were accompanied by the highest MDA content, more pronounced hepatopancreatic injury, and stronger TUNEL-positive signals. Negative enrichment of the Lysosome pathway further suggested that the cellular maintenance response observed at 15 ppt was not sustained at the higher salinity. The concurrent increase in the relative abundance of *Vibrio* also indicated a marked change in the intestinal bacterial community under the condition associated with the poorest tissue condition and survival.

From an aquaculture perspective, freshwater was the most suitable of the tested conditions for the long-term rearing of juvenile *E. j. sinensis* because it supported the greatest growth and production, whereas the survival advantage at 15 ppt did not compensate for the reduced growth and production. Where saline water is unavoidable, future studies should evaluate intermediate salinities between 0 and 15 ppt, gradual salinity adjustment, and nutritional strategies that maintain digestive capacity and energy supply to determine whether they improve growth without reducing survival. Salt-tolerant families could be screened according to their ability to maintain both growth and survival with limited hepatopancreatic damage. MDA, SOD, CAT, T-AOC, and serum ALT and AST activities may provide a candidate biochemical panel for this evaluation, supplemented by digestive enzyme activities. ACP, AKP, LZM, and GSH-Px may be less suitable as standalone indicators because their responses were compensatory or non-linear and should therefore be interpreted together with the proposed panel.

These integrated findings should nevertheless be interpreted in light of the experimental design. The biochemical, transcriptomic, metabolomic, and qRT-PCR analyses were based on three independent tank-level pooled biological replicates per treatment. This limited replication reduces the statistical power to detect small treatment effects and increases uncertainty in effect estimates. In addition, pooling tissues within each tank precludes the assessment of individual-level variation. Therefore, the biochemical and molecular results should be interpreted as treatment-associated patterns at the tank level rather than as individual-level responses. Although the concordance among the histological, biochemical, and multi-omics results supports the internal consistency of the main findings, further validation using a larger number of independent tanks and individual-level sampling is warranted. The intestinal microbiota analysis had an additional sampling limitation. Although the five cross-tank composite samples per treatment were prepared from non-overlapping sets of crabs, every composite contained material from the same three replicate tanks. Consequently, tank identity was not retained, and tank-specific microbial variation could not be estimated. The observed differences therefore represent treatment-associated patterns among the composite community profiles rather than five independent tank-level responses. Future studies should prepare intestinal microbiota samples separately within each independent tank to permit tank-level statistical inference.

## 5. Conclusions

Long-term rearing salinity had distinct effects on the performance and health of juvenile *E. j. sinensis*. Under the present experimental conditions, crabs reared at 0 ppt showed the greatest growth and total production and the least pronounced hepatopancreatic alterations, indicating the best overall rearing outcome among the tested salinities. Although 15 ppt resulted in the highest survival rate, it also produced the lowest individual growth and total production. This treatment therefore represented a survival-growth trade-off rather than a generally favorable condition for long-term production. Crabs reared at 30 ppt had the lowest survival rate and showed the most pronounced hepatopancreatic lesions, TUNEL-positive signals, lipid peroxidation, and reductions in overall antioxidant capacity. Transcriptomic and metabolomic analyses identified Lysosome and Tyrosine metabolism as prominent salinity-responsive pathways. Salinity was also associated with variation in the intestinal bacterial community profiles, with the relative abundance of *Vibrio* tending to increase and that of *Dysgonomonas* tending to decrease as salinity increased. Overall, neither saline treatment provided an advantage over freshwater when growth, production, and tissue condition were considered together. These findings demonstrate that the suitability of a salinity regime for the long-term culture of juvenile *E. j. sinensis* should be evaluated using survival, growth, production, and health-related indicators rather than survival alone.

## Data Availability

The raw data have been uploaded to the China National Center for Bioinformatics, and the accession number are PRJCA044388 and PRJCA044410.

## Supplementary Materials

The following supporting information can be downloaded at: https://www.mdpi.com/article/10.3390/ani16203192/s1, Figure S1: Morphology and morphometric measurement of juvenile Chinese mitten crab (*Eriocheir japonica sinensis*); Figure S2: Orthogonal partial least-squares discriminant analysis (OPLS-DA) score plots for the 0 ppt vs 15 ppt, 0 ppt vs 30 ppt, and 15 ppt vs 30 ppt comparisons; Figure S3: Dynamic analysis of the difference in metabolite content in three comparisons; Figure S4: Correlation between DAMs and DEGs in hepatopancreas. (A) Two-way orthogonal partial least squares (O2PLS) model (Red represents DEGs and blue represents DAMs; Figure S5: Nine quadrants of DAMs and DEGs identified from 0 ppt vs 15 ppt, 0 ppt vs 30 ppt, and 15 ppt vs 30 ppt; Figure S6: Correlation between DAMs and DEGs in hepatopancreas. (A). The first 30 KEGG pathway bubble charts enriched by transcriptomics and metabolomics; Figure S7: Intergroup variation of intestinal microbiota of juvenile *E. j. sinensis*, linear discriminant analysis effect size (LEfSe) cladogram; Figure S8: Intergroup variation of intestinal microbiota of juvenile *E. j. sinensis*, linear discriminant analysis (LDA) score of the abundance of taxa; Figure S9: Genus level correlation network analysis; Table S1: Types and nutrient components of compound feeds (%, dry matter); Table S2: Kits used for enzyme activity assay in this study; Table S3: Primer sequences used for qRT-PCR validation; Table S4: Quality control metrics of RNA sequencing for hepatopancreas samples; Table S5: Transcriptome analysis statistical table of DEGs.

## Abbreviations

ACE, abundance-based coverage estimator; ACP, acid phosphatase; AKP, alkaline phosphatase; Alb, albumin; ALT, alanine aminotransferase; AMPK, AMP-activated protein kinase; AMS, amylase; ANOVA, analysis of variance; AST, aspartate aminotransferase; BCA, bicinchoninic acid; bp, base pair; CAT, catalase; cDNA, complementary DNA; Ct, cycle threshold; DAM, differentially accumulated metabolite; DAPI, 4′,6-diamidino-2-phenylindole; DEG, differentially expressed gene; DNA, deoxyribonucleic acid; FDR, false discovery rate; FoxO, forkhead box O; FPKM, fragments per kilobase of transcript per million mapped fragments; GC, guanine–cytosine; GO, Gene Ontology; GSEA, gene set enrichment analysis; GSH-Px, glutathione peroxidase; H&E, hematoxylin and eosin; HSD, honestly significant difference; IACUC, Institutional Animal Care and Use Committee; KEGG, Kyoto Encyclopedia of Genes and Genomes; KGML, KEGG Markup Language; LC-MS, liquid chromatography–mass spectrometry; LC-QTOF, liquid chromatography coupled with quadrupole time-of-flight mass spectrometry; LDA, linear discriminant analysis; LEfSe, linear discriminant analysis effect size; LPS, lipase; LZM, lysozyme; MDA, malondialdehyde; NES, normalized enrichment score; NMDS, non-metric multidimensional scaling; O2PLS, two-way orthogonal partial least squares; OPLS-DA, orthogonal partial least squares discriminant analysis; OTU, operational taxonomic unit; PBS, phosphate-buffered saline; PCA, principal component analysis; PCoA, principal coordinate analysis; PCR, polymerase chain reaction; ppt, parts per thousand; Q^2^Y, cross-validated predictive ability of the model; Q30, percentage of bases with a Phred quality score of at least 30; QC, quality control; QIIME2, Quantitative Insights Into Microbial Ecology 2; qPCR, quantitative polymerase chain reaction; qRT-PCR, quantitative real-time polymerase chain reaction; QTOF, quadrupole time-of-flight; R^2^X, proportion of variation in the predictor variables explained by the model; R^2^Y, proportion of variation in the response variables explained by the model; RDA, redundancy analysis; RMSEE, root mean square error of estimation; RNA, ribonucleic acid; RNA-seq, RNA sequencing; ROS, reactive oxygen species; rRNA, ribosomal RNA; SE, standard error; SGR, specific growth rate; SHOU, Shanghai Ocean University; SOD, superoxide dismutase; SPSS, Statistical Package for the Social Sciences; T-AOC, total antioxidant capacity; TP, total protein; TUNEL, terminal deoxynucleotidyl transferase dUTP nick-end labeling; VIP, variable importance in projection; WGR, weight gain rate.
