# Supplementary material for: Effects of Long-Term Salinity Exposure on Growth, Survival, Hepatopancreatic Health, and Intestinal Microbiota in Juvenile Chinese Mitten Crabs (Eriocheir japonica sinensis)
Source: Animals (Basel). 2026 Oct 11;16(20):3192. doi: 10.3390/ani16203192 (PMC13700658; doi:10.3390/ani16203192)
Supplement: Supplementary file 1 [file animals-16-03192-s001.zip › animals-4568650-supplementary.pdf]

Supplementary Figures

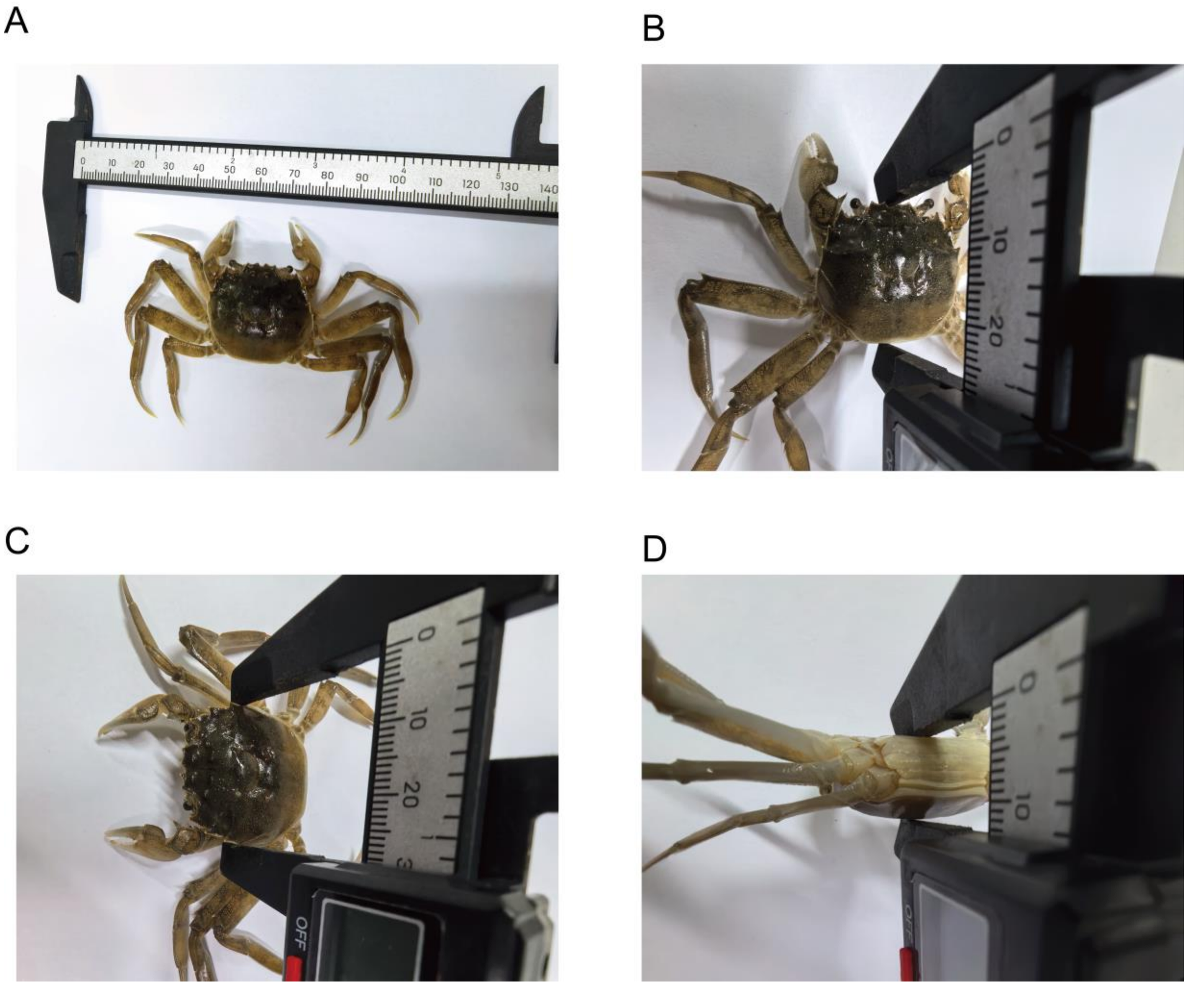

Figure S1. Morphology and morphometric measurement of juvenile Chinese mitten crab (*Eriocheir japonica sinensis*). (A) Representative photograph of a juvenile crab. (B-D) Measurement of body length (B), body width (C), and body height (D) using a digital vernier caliper. In this study, body length was defined as the distance from the median notch between the inner frontal teeth to the posterior margin of the carapace. Body width was defined as the maximum transverse distance between the outermost lateral margins of the carapace. Body height was defined as the maximum dorsoventral distance from the dorsal surface of the carapace to the ventral surface of the folded abdomen. All dimensions were recorded in millimeters.

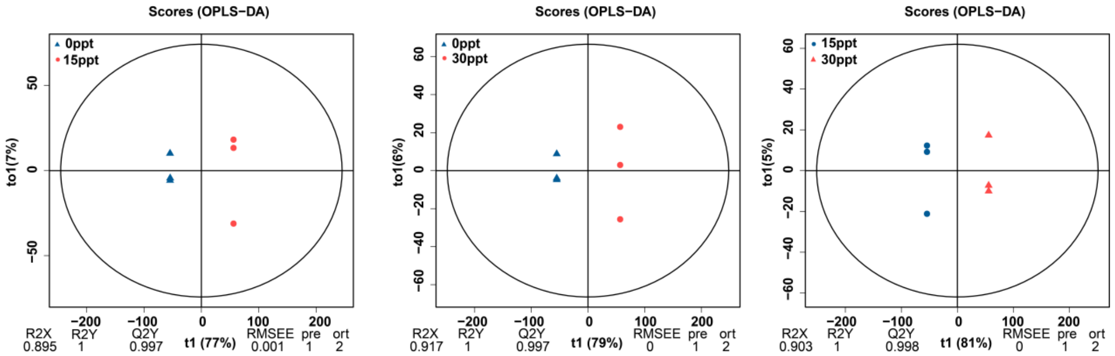

Figure S2. Orthogonal partial least-squares discriminant analysis (OPLS-DA) score plots for the 0 ppt vs 15 ppt, 0 ppt vs 30 ppt, and 15 ppt vs 30 ppt comparisons. The R^2X, R^2Y, and Q^2Y values are shown below each plot.

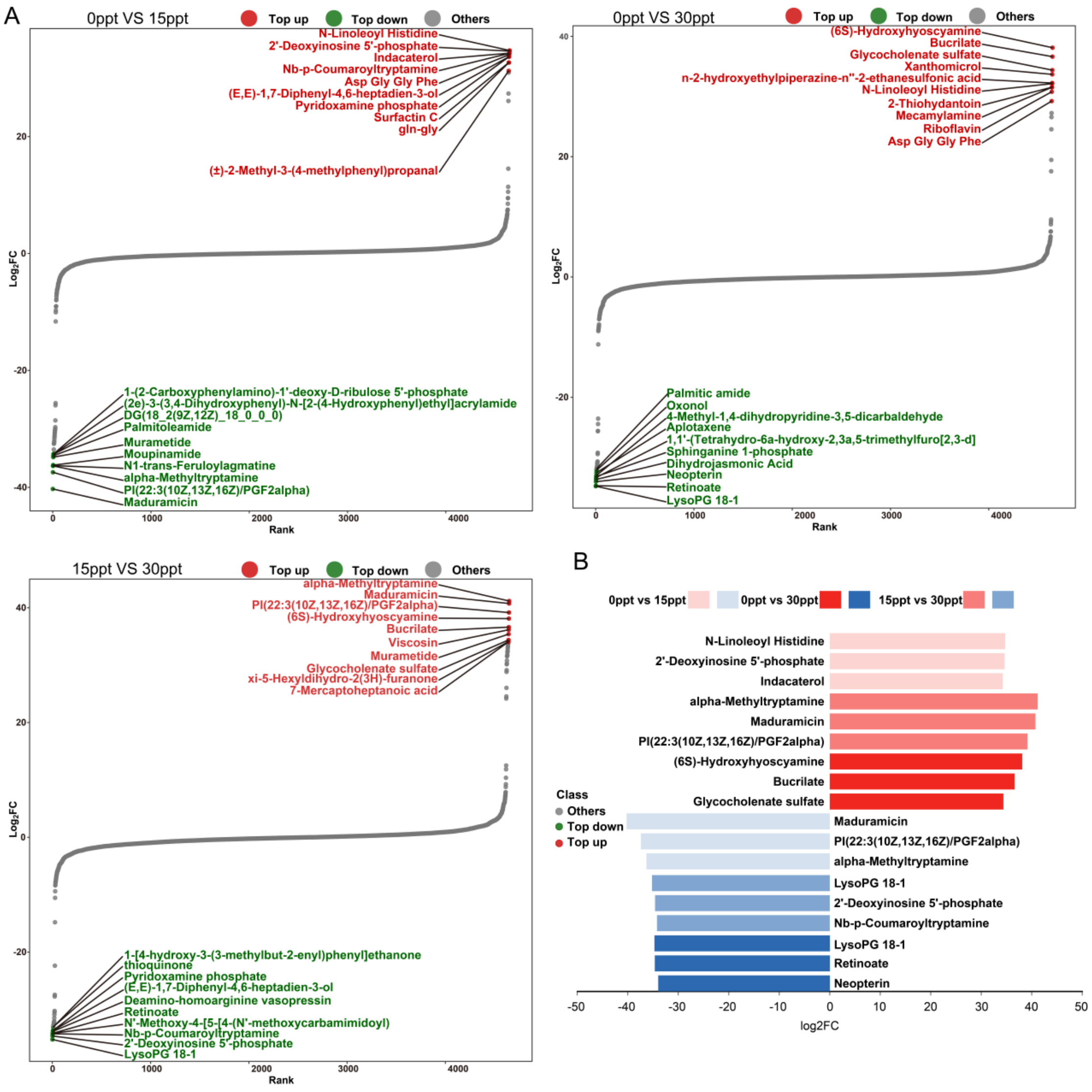

Figure S3. Dynamic analysis of the difference in metabolite content in three comparisons. **(A)**. Up-regulation and down-regulation of the top 10 metabolites, green dots represent down-regulation, and red dots represent up-regulation. **(B)**. Up-regulation and down-regulation of the top three DAMs, red for up-regulation, blue for down-regulation.

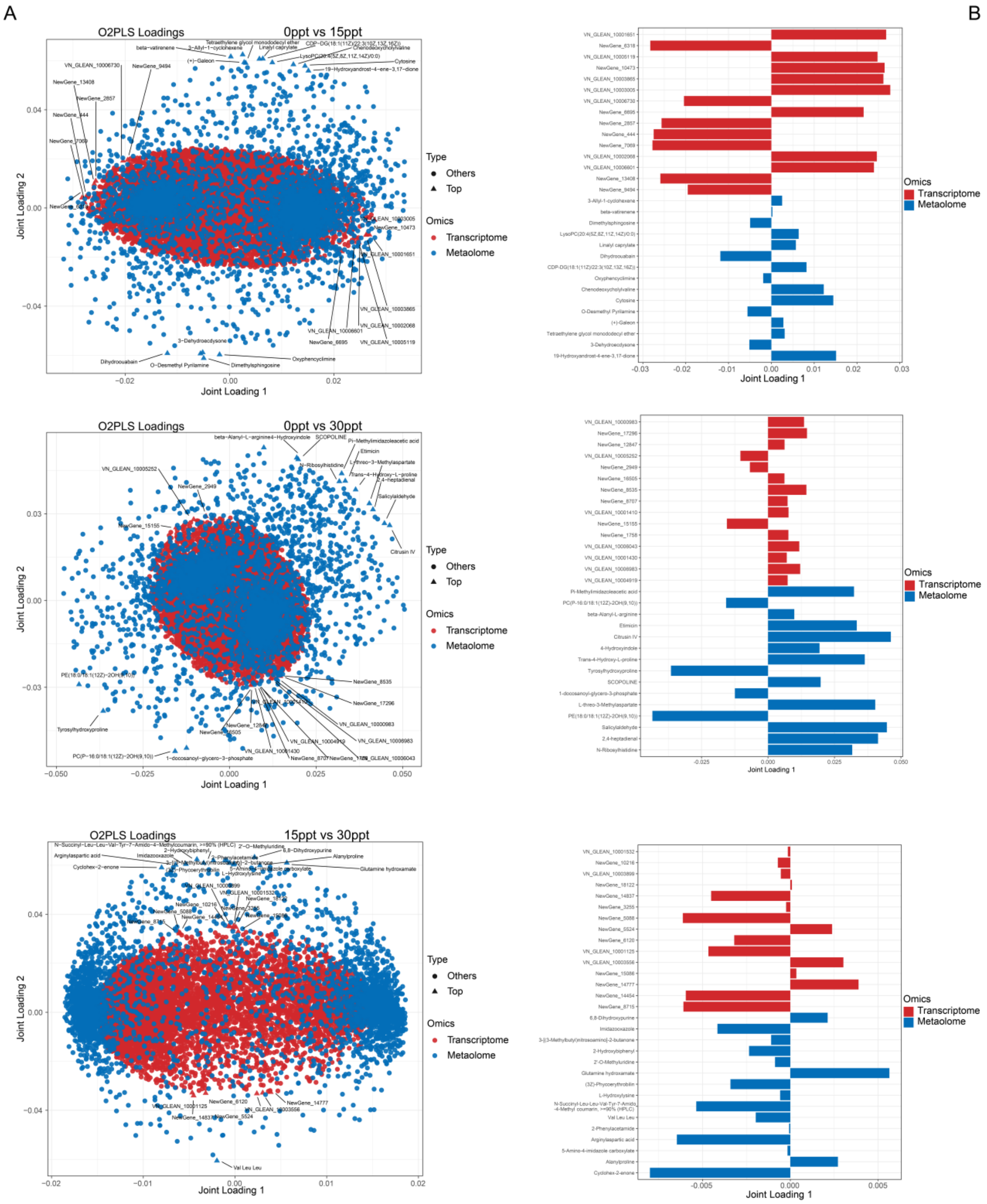

Figure S4. Correlation between DAMs and DEGs in hepatopancreas. **(A)** Two-way orthogonal partial least squares (O2PLS) model (Red represents DEGs and blue represents DAMs. The abscissa ordinate represents the combined load value. The load value length top 15 DAMs/DEGs are represented as Top, represented by solid triangles, and other DAMs/DEGs are represented as Others, represented by solid dots.) **(B)**. The top 15 DAMs/DEGs in the length of load value. The red column represents genes, and the blue column represents metabolites.

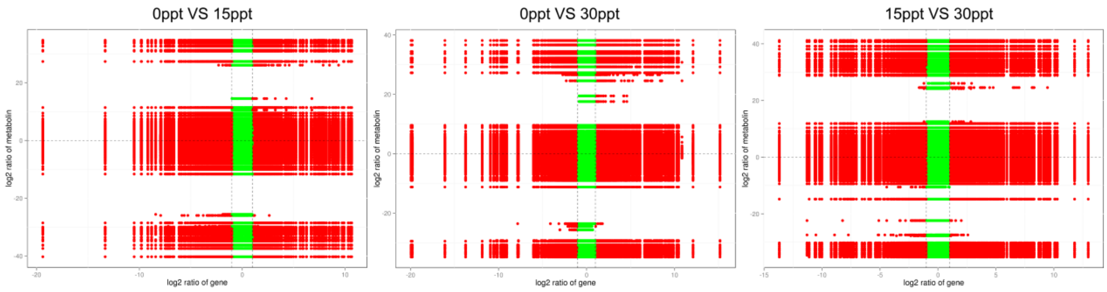

Figure S5. Nine quadrants of DAMs and DEGs identified from 0 ppt vs 15 ppt, 0 ppt vs 30 ppt, and 15 ppt vs 30 ppt.

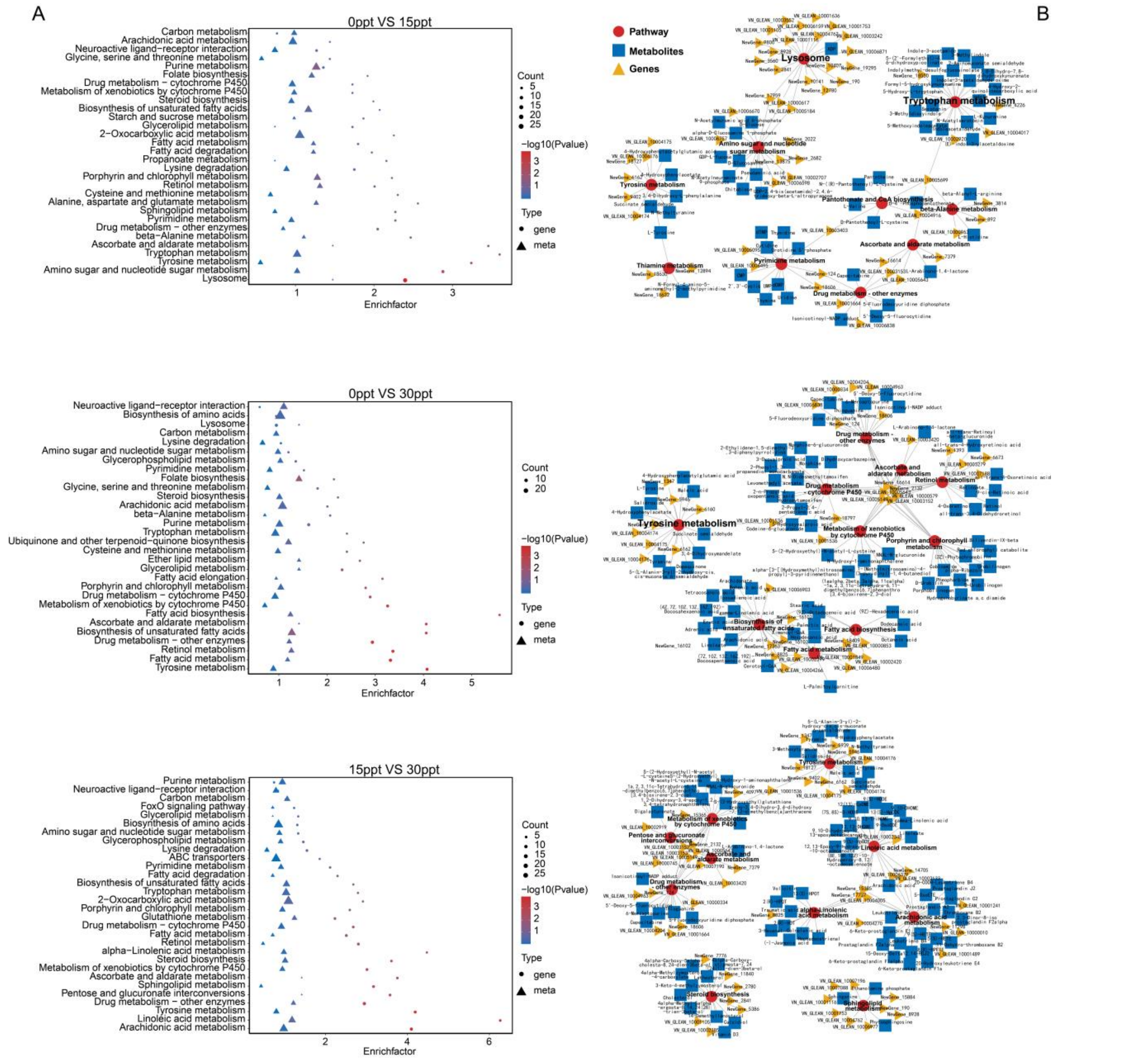

Figure S6. Correlation between DAMs and DEGs in hepatopancreas. **(A)**. The first 30 KEGG pathway bubble charts enriched by transcriptomics and metabolomics. The abscissa represents the enrichment factor of this pathway in different omics, and the ordinate represents the name of KEGG pathway. The gradual change of red to blue represents the significant change of enrichment from high to low, which is expressed by *P*-value. The shape of bubbles represents different omics, the circle represents transcription group, and the triangle represents metabolism group. The bubble size represents the number of differential metabolites or genes, and the larger the number, the larger the point. **(B)**. KEGG Markup Language (KGML) of top 10 KEGG pathway. Red circles represent KEGG pathway, blue squares represent

metabolites, and yellow triangles represent genes. Color represents the raw enrichment *P*-value. No multiple-testing correction was applied.

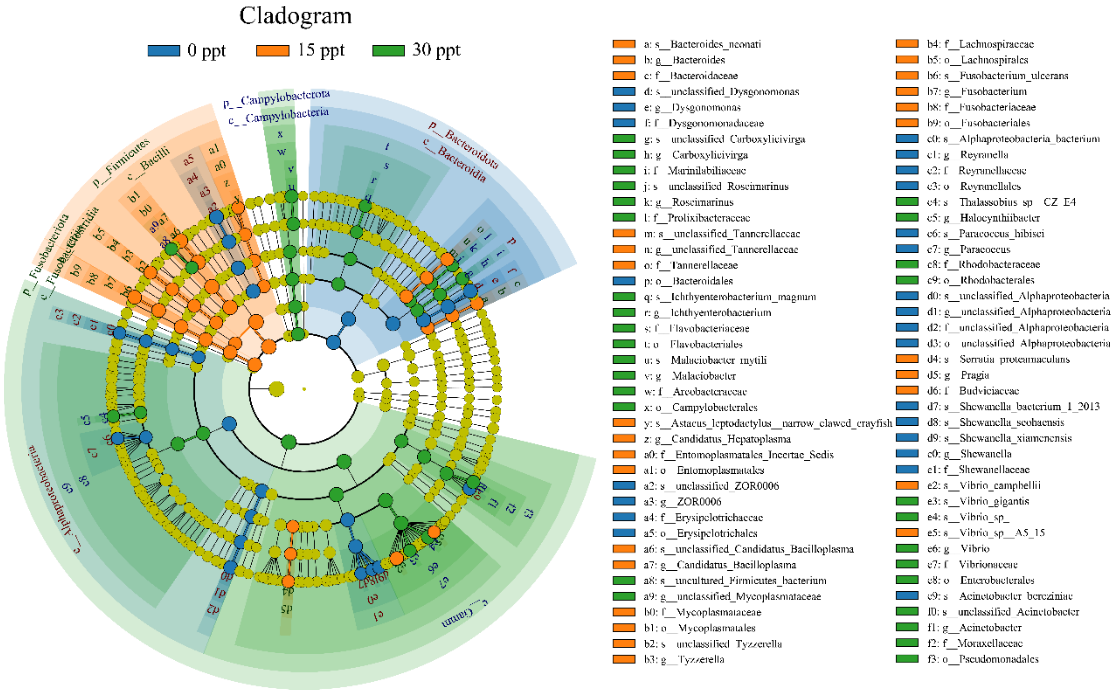

Figure S7. Intergroup variation of intestinal microbiota of juvenile *E. j. sinensis*, linear discriminant analysis effect size (LEfSe) cladogram.

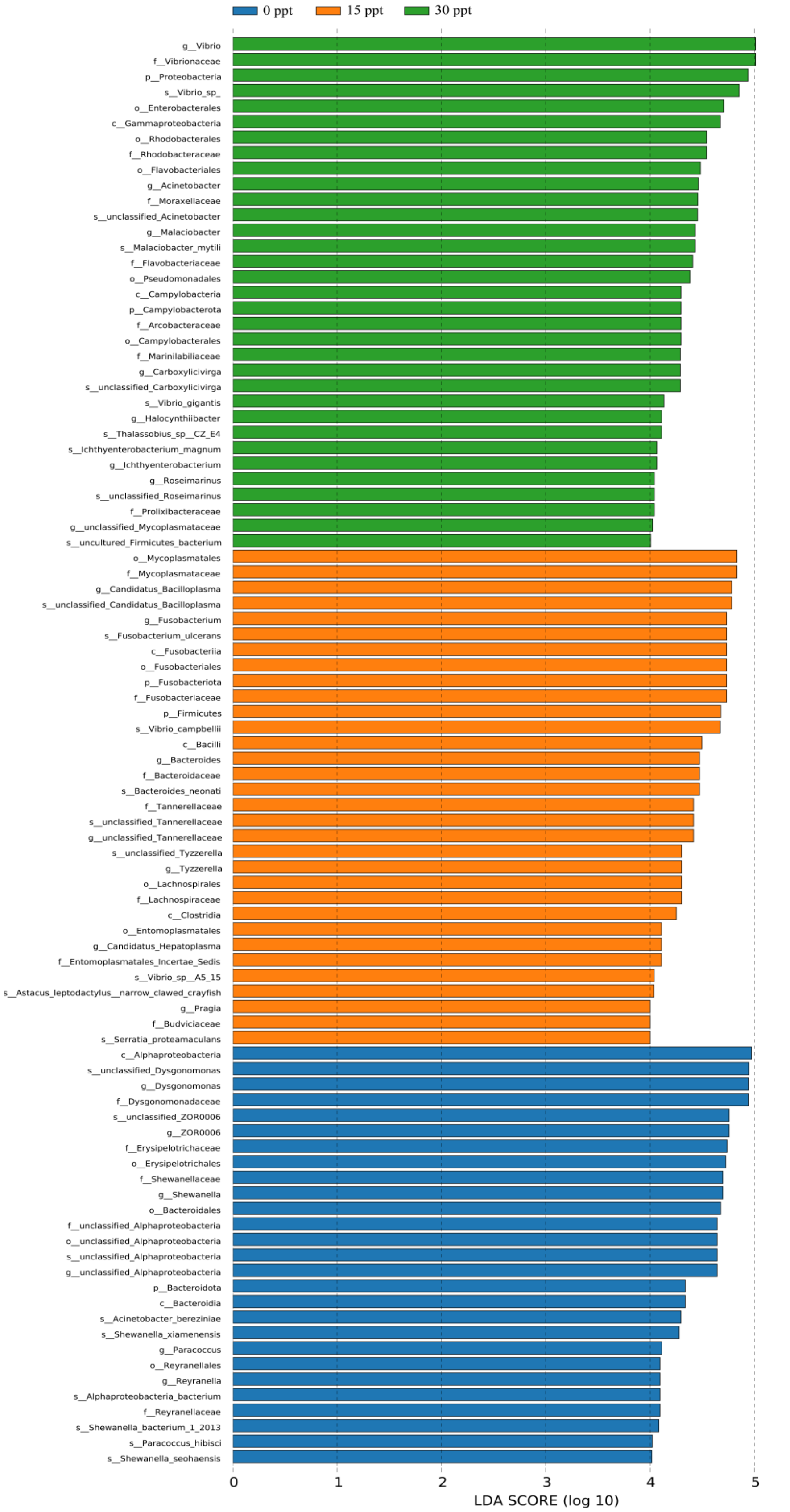

Figure S8. Intergroup variation of intestinal microbiota of juvenile *E. j. sinensis*, linear discriminant analysis (LDA) score of the abundance of taxa.

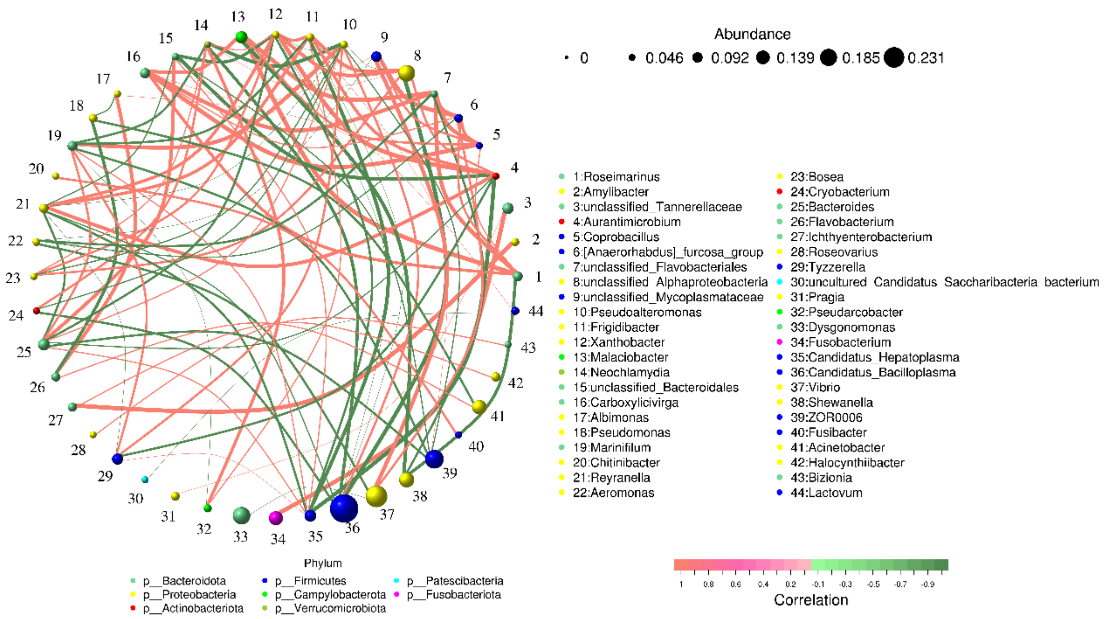

Figure S9. Genus level correlation network analysis. Circles represent species, circle size represents average abundance of species. Lines represent correlation between two species; line thickness represents correlation strength. Line color: red represents positive correlation, green represents negative correlation.

Table S1. Types and nutrient components of compound feeds (%, dry matter).

Feeding time	Moisture (%)	Crude protein (%)	Crude lipid (%)	Crude Ash (%)	Lysine (%)	Total phosphorus (%)	Diameter (mm)
May - Jun	10	42	6	15	2.3	1	0.1
Jul - Aug	10	42	6	15	2.3	1	0.3
Sep - Dec	10	42	4	15	2.5	1.2	1.0

Table S2. Kits used for enzyme activity assay in this study.

Enzyme	Item No
Acid phosphatase (ACP)	A060-1-1
Alkaline phosphatase (AKP)	A059-1-1
lysozyme (LZM)	A050-1-1
lipase (LPS)	A054-2-1
trypsin	A080-2-2
pepsin	A080-1-1
amylase (AMS)	C016-1-2
alanine aminotransferase (ALT)	C009-2-1
aspartate aminotransferase (AST)	C010-2-1
albumin (Alb)	A028-2-1
Catalase (CAT)	A007-1-1
Glutathione peroxidase (GSH-Px)	A005-1-2
Total superoxide dismutase (SOD)	A001-3-1
Total antioxidant capacity (T-AOC)	A015-2-1
Malondialdehyde (MDA)	A003-1-1
Total protein (TP)	A045-4-2

Table S3. Primer sequences used for qRT-PCR validation.

Primer names	Sequences (5′ - 3′)	Product size (bp)	GeneBank accession number	Annealing temperature (°C)	Primer efficiency
β-actin-F	GCATCCACGAGACCACTTACA	266	HM143924.1	54.4	99.36%
β-actin-R	CTCCTGCTTGCTGATCCACATC			57.2	
PHC-F	TCAAGCCCCACTACT	131	XM_050847176.1	42.8	94.84%
PHC-R	GGCGACTCTCTGTTC			45.5	
MOXD1-F	TGAGGGTTATCTGGG	191	XM_050881063.1	42.8	93.34%
MOXD1-R	GACGGTGTCTGTGTG			45.5	
MIF-F	CTGTGCTGGAGGTGT	167	XM_050840670.1	45.5	97.50%
MIF-R	GCAAGGTTCAGTGGT			42.8	
HGD-F	AGCAGGAGATGGATT	135	XM_050874410.1	40.0	104.44%
HGD-R	CGTCGCTGTTGTAGA			42.8	
ALDH3A1-F	GGAACCCTACGGAGT	111	XM_050838408.1	45.5	102.78%
ALDH3A1-R	GGCTTGATGACCACC			45.5	
AP1G1-F	TTCTGTATGGCG	114	XM_050883115.1	36.0	93.68%
AP1G1-R	GTGAAGCGTGGT			38.0	
TINAGL1-F	GTGATGTTGGGA	130	XM_050863564.1	36.0	101.29%
TINAGL1-R	TGGTGCTCTGAT			36.0	
lip-F	GGCGGAACAGAG	196	XM_050879991.1	40.0	97.64%
lip-R	GCAGAGGAGGCA			40.0	
ARSB-F	TGACGGGCTACT	258	XM_050857856.1	38.0	97.13%
ARSB-R	ACCTCTGCGAAG			38.0	
ARSA-F	GGTGTGTTTTTT	140	XM_050836227.1	32.0	105.01%
ARSA-R	TCTCATTTTCCA			32.0	
AP3B2-F	GGTAGCGAAGG	157	XM_050832079.1	36.0	98.53%
AP3B2-R	CTGGAAGGTGG			36.0	
NT1-F	TTCTGGCGTGCG	154	XM_050835918.1	40.0	98.02%
NT1-R	TCGGGGTCCTCG			42.0	
SLC17A5-F	CCAACTCCACCCCTT	112	XM_050867314.1	45.5	95.08%
SLC17A5-R	TCCCACAGCACGAAC			45.5	
CTNS-F	CCTCCTCGTCCTGCT	154	XM_050872465.1	48.2	95.61%
CTNS-R	ACTGTGGCGTTCTCG			45.5	
SMPD1-F	GCACTCTCCCGT	120	XM_050868977.1	40.0	104.56%
SMPD1-R	GGTCCCATCCAC			40.0	
MAN2B1-F	TGAATGACGAAGCCT	165	XM_050857678.1	40.0	99.96%
MAN2B1-R	CCATCTGTGCGAAGA			42.8	
NAGA-F	GCTGGGGCTGAAGTT	153	XM_050872395.1	45.5	99.12%
NAGA-R	TCGGGGGTGGAGTAG			48.2	
PTCH2-F	ATGGTGTGCGTG	136	XM_050834778.1	38.0	107.25%
PTCH2-R	GGAAGTTTTCGG			36.0	
CTSD-F	CTGGGGAGAATA	166	MG547692.1	36.0	96.73%
CTSD-R	TACCGATGGAGA			36.0	
GUSB-F	TGAGGAAGAGTG	122	XM_050840999.1	36.0	98.37%
GUSB-R	TGGAACATAGAA			32.0	
GALNS-F	AGAGCGGAAGGAGTA	101	XM_050859517.1	42.8	106.25%
GALNS-R	GCCCAGTAGAGGAAA			42.8	
GLB1L2-F	CCTCAACGACAAGCC	189	XM_050874698.1	45.5	102.13%
GLB1L2-R	ATCATCTCCCCAAGC			42.8	

Table S4. Quality control metrics of RNA sequencing for hepatopancreas samples.

Samples	Clean reads	Clean bases	GC Content	% ≥ Q30
0 ppt-1	19,636,989	5,868,795,710	49.66%	93.56%
0 ppt-2	20,318,413	6,069,093,153	49.51%	93.60%
0 ppt-3	20,756,770	6,201,249,649	49.40%	93.33%
15 ppt-1	20,673,170	6,171,984,736	49.08%	93.28%
15 ppt-2	20,162,525	6,023,466,891	48.52%	93.68%
15 ppt-3	22,886,789	6,845,650,825	48.72%	93.56%
30 ppt-1	21,448,951	6,392,803,218	49.21%	93.54%
30 ppt-2	26,395,763	7,869,011,085	49.20%	93.41%
30 ppt-3	21,080,439	6,284,964,098	49.09%	93.41%

Table S5. Transcriptome analysis statistical table of DEGs.

DEG Set	DEG number	Upregulated	Downregulated
0 ppt vs 15 ppt	1,019	467	552
0 ppt vs 30 ppt	1,097	535	562
15 ppt vs 30 ppt	1,095	517	578
